# Resolution Pharmacology: State-of-the-art and therapeutic landscape

**DOI:** 10.1016/j.pharmr.2025.100097

**Published:** 2025-10-08

**Authors:** Mauro Perretti, Trinidad Montero-Melendez

**Affiliations:** 1 The William Harvey Research Institute, Queen Mary University of London, Charterhouse Square, London, United Kingdom; 2 Centre for inflammation and Therapeutic Innovation, Queen Mary University of London, Charterhouse Square, London, United Kingdom

## Abstract

In 2015, we coined the term “Resolution Pharmacology” to indicate how the biology of the resolution of inflammation could, and should, be harnessed to produce novel therapeutics. Here we update these concepts and discuss the most recent developments in this innovative field of pharmacology.

We begin by discussing how the inflammatory response is lifesaving through the engagement, expression, and function of several mediators, which have been labeled as proinflammatory or proresolving mediators. In reality, they act in concert and regulate each other in a fully integrated fashion, so that the notion between inflammation onset and inflammation resolution, as 2 distinct phases, is mainly didactic and temporal. Moreover, the observation that the inflammatory reaction that our body mounts always, or nearly always, resolves indicate that inflammation resolution is a robust process. What remain to be addressed, though, is how to harness the biology of acute resolving inflammation so that innovative therapeutic options can be offered for the clinical management of chronic nonresolving inflammation.

The holistic view of physiological inflammation, and its disruption in pathology, comes with implications in relation to the application of Resolution Pharmacology. We predict resolution-based drugs will work better in presence of a florid inflammatory status, which would augment expression of resolution targets. We conclude by proposing a renovated focus on endogenous tissue-protective regenerative pathways by specific targets for drug development programs: Resolution Pharmacology remains an untapped opportunity for the pharmaceutical industry.

**Significance Statement:**

The process of the resolution of inflammation represents an integral part of the whole acute inflammatory response. A florid inflammatory reaction ensures proper engagement of resolution mechanisms. Dysregulation in resolution mechanisms can lead to disease. Conversely, harnessing resolution can offer therapeutic guidance to develop medicines that are disease independent, broadening their potential. There is ongoing intensive clinical development in this area. Proresolving drugs will be patient centric in their pharmacology and would promote natural processes of healing and repair.

## I Introduction

Ten years ago, we wrote a position review where we coined the term Resolution Pharmacology.^1^ With it, we intended to stress the great potential for translating the fundamental biology of the resolution phase of the inflammatory response to guide innovative approaches to drug discovery. We reasoned that the industry had ignored 50% of the functions set out in our body to resolve inflammation, as all anti-inflammatory drugs on the market had been developed by determining their ability to block or inhibit the actions of specific proinflammatory mediators. A second concept presented in the 2015 review was the prediction that therapeutics based on the way (mediators, targets, pathways) our body resolves acute inflammation would be devoid of substantial secondary effects and therefore be characterized by a better patient adoption, all in all delivering a personalized medicine treatment.^1^ We now wish to provide an update on both our understanding of the fundamental mechanisms of resolution and the approaches taken in drug discovery programs based on them.

The last decade has witnessed a series of discoveries that have augmented our understanding of the inflammatory response, with 2 relevant appreciations. First, the inflammatory reaction is a fundamental response of our body and as such it should not be demonized, rather the contrary as it is lifesaving. There has been a better characterization of how the body responds to encounters with xenobiotics, infective agents, and traumatic insults. As such, we distinguish acute inflammation—which may last 3–7 days postinsult—from chronic inflammation which last months if not years. The second concept that emerged is that virtually every disease, we humans may suffer from, contains an inflammatory component. The “specific weight” of this component may vary depending on the disease in question; nonetheless, inflammatory mediators and targets are more often than not relevant from an etiopathogenic perspective. This concept implies that drugs that control specific inflammatory reactions can be beneficial in several disease settings. Classical “inflammatory diseases” are obviously arthritides and vasculitides, where the *inflammatory component is very high*; however, cardiovascular diseases are also brought about through a *substantial inflammatory component*, examples being atherosclerosis and cardiac inflammatory syndromes. Metabolic diseases also present a *reasonable inflammatory component* with the more recent awareness that even pathologies of the central nervous system like Alzheimer disease and epilepsies contain a *certain degree of inflammation* in their fundamental pathogenic mechanisms. Such schematic view is often more complex as chronic diseases seldom derive from a single pathology as they are very often characterized by the presence of comorbidities; these include obesity/overweight, diabetes, and/or hypertension. Therefore, several and potentially different inflammatory pathways may operate in distinct tissues and organs, opening the possibility that distinct points of therapeutic intervention may exist, as discussed later. Ten years on, now we report on a fervid therapeutic landscape and predict that innovative drugs will be available in the near future both for patient benefit and to equip doctors with novel medicines for the clinical management of chronic diseases that affect our society.

## II The pillars of Resolution Pharmacology

**Fig. 1:**
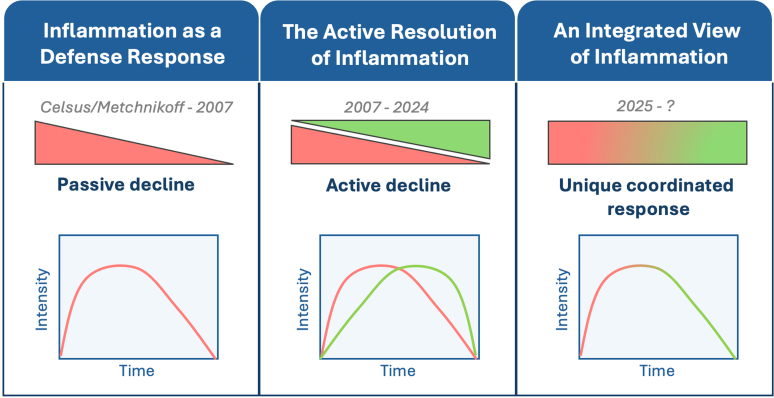
A new integrated view of the inflammatory response. Schematic view on the way our appreciation of the acute inflammatory reaction has developed over time. *Left panel*: Over several centuries great focus was given on the elucidation of the mechanisms and processes that cause inflammation. Identification of specific molecules that promote these processes, and receptors that enable such effects, has led to the development of drugs that act by blocking a given mediator’s actions, often through receptor antagonism. This long period of science considered the termination of the inflammatory reaction to be passive. *Middle panel*: A review in 2007 set the scene for clarifying the active nature of the second phase of the inflammatory reaction, the one then termed “resolution phase.” Since then, major research efforts from a larger number of groups have been made to identify proresolving mediators, elucidate the mechanisms that they exert (predominantly on immune cells), and describe the receptor targets activated to achieve these outcomes. *Right panel*: With the present review, we reason on the integrated profile of acute resolving inflammation, as well as on the evidence that florid, strong and efficient inflammatory response leads to termination and resolution, without off-target tissue damage. As such, the so-called proinflammatory and proresolving mediators are in fact just inflammatory mediators, fully interconnected in relation to their reciprocal expression and function. Based on the biology of these mediators, pharmacologists must devise therapeutic opportunities: so far, only approaches to block specific mediators have been translated to human benefit. Here we review the current efforts to mimic the properties of specific mediators for clinical benefit.

A review in 2007^2^ is indicated as the starting point for the concept of resolution of inflammation: this concept brings together a distinct cluster of mechanisms activated by mediators/receptors to terminate the inflammatory response. Serhan et al^2^ reasoned on the novel notion that the second phase of acute inflammation was active and not passive. From the initial investigations that have elucidated mechanisms of inflammation, eg, the process of white blood cell recruitment (Conheim) or phagocytosis of infectious agents (Metchinkoff), the focus of basic and clinical scientists has been to identify the major symptoms of inflammation (Celsus) and, mainly in the 20th century, study specific molecules responsible to promote if not induce both the humoral and cellular mechanisms of inflammation (Fig. 1, left panel).

During this long phase of research, the termination of the acute inflammatory response was considered to be passive and, essentially, set in motion by the absence (through catabolism or spontaneous inactivation) of the molecules responsible to mount the inflammatory response itself. The concept of *passive extinguishment* spawns a major pharmacological consequence: to control inflammation it would be sufficient to block the actions of molecules that promote it. Following the discovery of the antirheumatic properties of glucocorticoids and adrenocorticotrophin (ACTH),^3^ anti-inflammatory drug development programs yielded nonsteroidal anti-inflammatory drugs to inhibit cyclo-oxygenase,^4^ antagonists to histamine and, more recently, biologics to block specific cytokines or antagonize their receptors.^5^ In the 2007 review,^2^ we offered a different perspective, whereby acute inflammation resolves actively through the function of proresolving pathways and the engagement of proresolving receptors (Fig. 1, middle panel). This novel notion was supported by the studies which showed a temporal switch between proinflammatory lipids like eicosanoids to proresolving lipids like lipoxins,^6^ as well as the augmented if not prolonged inflammatory reaction observed if specific endogenous molecules were absent or their effects blocked.

The *active nature* of the resolution of inflammation, which has been discussed in several reviews from the 2007 one, exhorts 2 major considerations. The first is linked to etiopathogenesis: although active resolution explains the tight control in time and space of the acute inflammatory response, its absence or defective engagement can at least contribute to the establishment of chronic inflammation. This implies that the large series of diseases that affect our societies may be due, at least in part, to inadequate engagement of proresolving pathways and mechanisms. The second is linked to pharmacology and, in essence, underpins the present review: with respect to the development of anti-inflammatory therapeutics, we have just used 50% of the biology of inflammation, ignoring a large section that is fundamental to ensure its natural termination. This second consideration implies that chronic diseases which, as said above, all present an inflammatory component, can benefit by application of resolution-based medicines or, equally relevant, specific clinical manifestations of these complex diseases can be better controlled through the application of resolution-based medicines. These 2 elements will be discussed later.

Herein, we propose a *third view* to schematize the acute inflammatory response, and this is presented in Fig. 1 (right panel). There is evidence for a close interlink between onset-to-peak and peak-to-end phases of inflammation: in reality it is a single phenomenon brought about from beginning to end by inflammatory and resolution mediators acting in concert. Earlier we indicated the study linking a switch between prostanoids and lipoxins.^6^ Several other studies have demonstrated the functional link between the previously indicated proinflammatory *versus* proresolving phase. Thus, mediators like tumor necrosis factor-*⍺* (TNF-*⍺*) promote expression of proresolving mediators like annexin A1 (AnxA1) and receptors like formyl peptide receptor 2 (FPR2).^7^ In addition, anti-TNF biologics are effective in patients affected from inflammatory bowel disease when their application leads to higher AnxA1 levels in tissue biopsies.^8^ Moreover, experimental data indicate that absence of proresolving mediators cannot simply lead to higher degree of acute inflammation or prolonged acute inflammation, but it can also lead to an initial inadequate expression of proinflammatory mediators. As an example, AnxA1 null mice display prolonged intestinal inflammation which does not resolve, while it had resolved in control animals.^9^ However, in settings of experimental sepsis, these animals produce lower levels of TNF-*⍺* and interleukin (IL)-1*β*1–2 hours after injection of lipopolysaccharide, although these cytokine expression levels remain very elevated at later time points, when in control animals had subsidized by then.^10^ In other words, absence of AnxA1 provokes a misfit inflammatory reaction from its start. These observations indicate the existence of a tonic role of AnxA1 which regulates the whole of the inflammatory response including the early release of proinflammatory cytokines presumably from macrophages. The same has been shown for influenza infection, where endogenous AnxA1 “prepares” dendritic cells to mount a good immune response and absence of this mediator leads to an inadequate and floppy response of the host once infected.^11^ In the same vein, there is evidence that proresolving mediators can activate immune cells and provoke responses that can be superficially indicated as proinflammatory. Application of lipoxin A_4_ (LXA_4_) to human neutrophils provokes release of reactive oxygen species (while it inhibits this response once applied to cells from patients).^12^ Similarly, AnxA1 addition to human monocytes promotes their chemotaxis, while inhibiting this cellular response if CCL-2 is applied as chemotactic agent.^13^

Two further notes are relevant here. The first one is that cellular responses, as these just mentioned, are not really indicative of inflammation *per se* but rather represent one of the multiple aspects of cell activation within the whole of the inflammatory response. The second one is that acute inflammation is lifesaving and highly beneficial for the host. Therefore, all its elements (factors, mediators, molecules, pathways, receptors, signaling cascade, and so forth) operate to ensure its perfect functioning and are indispensable to achieve the ultimate goal of protection and regaining of tissue or organ functionality. This is visualized in Fig. 1 (right panel) where acute inflammation is represented as a single line where the color of the line varies to indicate a predominance of one class of molecules and associated phenomena over time. So-called proinflammatory mediators kick in so-called proresolving mediators, and the latter are required from the start to ensure adequate production of the former: in this manner a full-blown inflammatory response resolves over time.^14^

Medzhitov^15^ has pioneered a similar view when discussing the existence of tissue settings required for homeostasis, and how diversion from these settings, either too high or too low, excludes homeostasis and leads to pathology. The underlying low-level inflammation that never blasts and never resolves is what becomes responsible for chronic inflammation or for the comorbidities associated with chronic diseases, as chiefly reported for low degree inflammation in the adipose tissue.^16^^,^^17^ Recent work demonstrated immune activation within the pericardial adipose tissue and how this is associated with cardiac inflammatory syndromes.^18^^,^^19^ Activation of inflammatory cells, like macrophages and mast cells, feed forward tissue-restricted alterations that contribute to the onset of pathology. This is an example of perturbation of the homeostatic circuits that lead to inflammation, with downstream alterations being due to loss of structure (eg, tissue damage), loss of function (eg, infective agent), or loss of control (eg, physiological alterations consequent to changes in temperature or nutrients).^20^

**Fig. 2:**
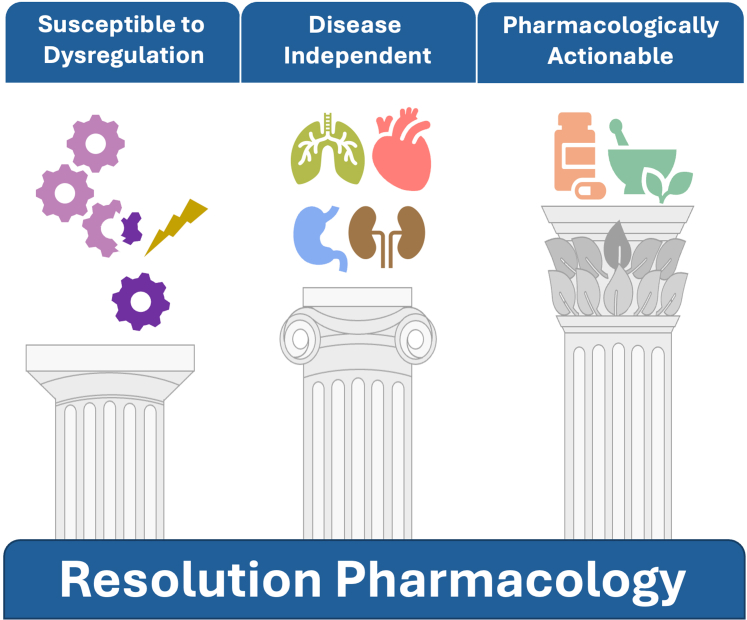
The pillars of Resolution Pharmacology. Over the centuries, the Greek architectonical style has developed as pictured with the different capitals on the Greek columns, shown here. The simplest or basic style is that of the *Doric* capital (column on the left) which was used from seventh century BC. We see it here to represent the fundamental biology of the inflammatory response, essentially the basis—in physiological settings—on how our body deals with changes off the equilibrium. This is achieved through a unique coordinated response typical of acute resolving inflammation. Building on this knowledge, and capital, the *Ionic* style (column in the middle) entered the scene around fifth century BC, with the volutes that offers more subtle style—then modified with the volute on the corner angling to ensure equal view from both sides. This “uniform” view is mirrored here by remarking the potential extensive application of resolution pathways to control several diseases, or specific elements of several chronic diseases. The most complex type of capital is the *Corinthian* one, stemmed from the town of Corinth around fourth to third century BC: it presents volutes, acanthus leaves, and a flower. In our “humble analogy,” we match the Corinthian capital with harnessing the knowledge of inflammation with the development of Resolution Pharmacology, and the multivaried way that this could be achieved, as discussed in this article. The Corinthian capital has enjoyed a much longer life and use, being used by the Romans as well as in other forms by several other cultures up to the 10th century AD.

The narrative discussed above provides the background onto which develop Resolution Pharmacology from the biology of resolution or as proposed, from the unique coordinated response that is represented by acute inflammation. Thus, the resolution response is *susceptible to dysregulation* (through absence, or too little or too much of a given mediator and its ensuing regulated pathways). In addition, resolution-based drugs will be applicable to multiple diseases, ie, their use will be *disease independent*: such a view is linked to the inherent inflammatory nature of virtually all human pathologies. Finally, resolution-based mechanisms, including mediators and targets, are *pharmacologically actionable*: we will discuss several ways in which Resolution Pharmacology can be delivered. Figure 2 presents these 3 concepts onto which we have structured the rest of this review.

## III Endogenous resolution mechanisms are susceptible to dysregulation

In the past, when the termination of an inflammatory response was understood as a passive process, it could only be conceived that persistence of inflammation indicated persistence of the proinflammatory insult. The acceptance of the active and regulated nature of resolution, however, shifted this view, as we now conceive that persistence of inflammation may also be the result of a failure of the endogenous proresolving mechanisms. This notion has been directly proposed and studied in various disease states. In rheumatoid arthritis (RA), it has been suggested that synovial fibroblasts remain in state of hyperactivation causing a continuous reciprocal activation with immune cells, resulting in a failure of resolution within the joints.^21^ This inability to resolve have been linked to stable epigenetics changes which lock fibroblasts in a permanent proinflammatory state.^22^ Then, fibroblasts have emerged as an important cellular target to promote resolution, using melanocortins^23^ or lipid mediators.^24^^,^^25^

AnxA1 null mice displayed higher inflammatory responses in settings of paw edema as well as inflammatory arthritis.^9^^,^^26^, ^27^, ^28^ In another form of joint arthritis provoked by monosodium urate crystals-induced gout in mice, mice lacking AnxA1 present with greater inflammation and delayed resolution, suggesting again that a failure of endogenous proresolving mediators can directly lead to or exacerbate disease.^29^ Similarly, absence of the AnxA1 receptor, *Fpr2*, is associated with exacerbated inflammation in the self-resolving serum transfer-induced arthritis model in mice^30^ and delayed resolution compared with wild-type mice. Mice lacking 12/15 lipoxygenase (*Alox15*^–/–^) display uncontrolled inflammation and more pronounced tissue damage in 2 models of experimental arthritis, effects associated with lower levels of proresolving LXA_4_.^31^ Such a protective role for this enzyme has been also shown in a model of instability-induced osteoarthritis (OA), with marked cartilage degeneration as compared with control mice.^32^ In human RA, it has been reported a deficient production of resolvin (Rv) D3 in plasma of patients compared with healthy volunteers, and consistently, low RvD3 levels were associated with delayed resolution of arthritis in mice.^33^

Atherosclerosis is another area in which deficient resolution has been linked to disease. Inflammation is a main feature of atherosclerosis, in addition to dyslipidemia. Although typical treatments like statins regulate the latter, the inflammatory process persists contributing to the increased risk of cardiovascular events, despite controlled low-density lipoprotein levels.^34^ It has been proposed that an imbalance on the production of proresolving mediators like resolvin RvD1 and proinflammatory molecule leukotriene B_4_ (LTB_4_) determines whether atherosclerosis will resolve or not.^35^ Defective efferocytosis have been reported in atherosclerosis due to increased expression of “don’t-eat-me” signal CD47 on apoptotic cells,^36^ leading to the accumulation of dead cells within the atherosclerotic lesions potentiating vascular inflammation and risk of plaque rupture. Previous work already demonstrated the regulatory properties of type 1 receptor tyrosine kinase (MerTK).^37^ Mutation or deficiency of this receptor within plaque macrophages increased accumulation of apoptotic cells or promoted an expansion of the necrotic ones, respectively. This study provided in vivo relevance to previous in vitro experimentation conducted by Ira Tabas et al.^38^

Similarly to defective efferocytosis leading to impaired resolution, we recently proposed that defective clearance of senescent cells also represents a failure of resolution.^39^ Similarly to apoptotic cell clearance, an efficient and timely clearance of senescent cells is required to prevent the detrimental effects of a persistent senescence-associated secretory phenotype released by senescent cells, which will prevent the restoration of tissue homeostasis. Of interest, senescent macrophages are not fully able to engulf apoptotic cells, due to the higher expression of do-not-eat me signals, like CD24 and CD47: this favors formation of the atherosclerotic plaque.^40^ Relevant to this review, this defect can be rectified by RvD2 if associated with reduced levels of CD24 and CD47. In any case, we should be aware that senescent cells are far from being “quiet” as they release a proinflammatory secretome containing cytokines, chemokines, proteases, growth factors, and more. If not cleared, for example due to an aged immune system,^41^ their accumulation in tissues leads to inflammation and tissue damage.

Other cardiovascular diseases have also been associated with defective resolution like myocardial infarction or atrial fibrillation.^42^ RvD1 can impact on the outcomes after myocardial infarction.^43^ This study made an interesting distinction between early versus late treatment with the lipid mediator: although early delivery of the compound (at infarction onset) reduced tissue injury and atrial remodeling, late delivery (day 7 postinfarction) reduced atrial remodeling and atrial fibrillation. The latter changes occurred without ventricular protection. Furthermore, genetic variants on genes involved in the biosynthesis of lipid mediators increase the risk of myocardial infarction and stroke.^44^ Mice lacking *Mc3r* receptor present increased immune cell trafficking and production of proinflammatory mediators in a model of ischemia reperfusion.^45^ Proresolving lipid mediators protect against cardiac damage associated with heart failure, with a cellular mechanism associated to modulation of leukocyte trafficking and their lifespan once recruited to the hearts.^46^^,^^47^ Halade et al^46^ demonstrated the nonredundant role of proresolving lipid mediators generated through the 5 lipoxygenase (LOX) and 12/15 LOX pathways. The same group demonstrates the role of a specific proresolving receptor; hence absence of *Fpr2* in mice is associated with leukocyte dysfunction and enhanced cardiovascular inflammation.^48^

Mice that lack the adenosine A2a receptor exhibit impaired efferocytosis and altered inflammatory responses due to inadequate engagement of resolution pathways.^49^ Furthermore, melanocortin 1 receptor (MC_1_) null mice develop intestinal inflammation^50^ and FPR2 null animals display a similar phenotype upon challenge.^51^ Finally for these examples, mice lacking the MAS1 receptor (product of the MAS1 proto-oncogene) are resistant to the beneficial effects of the heptapeptide angiotensin1–7 (Ang1–7) demonstrated in infections; see references^52^^,^^53^ for recent studies and an updated review by Tavares et al.^54^

It is important to highlight that the practical application of Resolution Pharmacology, does not require necessarily defective proresolving mechanisms or mediators in the first place, ie, Resolution Pharmacology is not a “corrective” approach, but rather it can be used to control disease progression by potentiating the natural mechanisms available within the patient. For example, the melanocortin receptor MC_1_ may be fully functional in patients with scleroderma. However, its pharmacological targeting using an agonist has shown promise in treating this condition.^55^

## IV Resolution Pharmacology is disease independent

### A Resolution as a universal drug target

Classical drugs targeting inflammation, like nonsteroidal anti-inflammatory drugs or glucocorticoids, are nonspecific and are used for a broad range of diseases and conditions, like injury, trauma, surgeries, back pain, sunburns, sprains, or more complex pathologies associated with chronic inflammation like autoimmune diseases or immune-mediated inflammatory conditions.^56^ To a great extent, the cellular and molecular inflammatory processes occur in a similar manner: initiation phase characterized by a strong vascular response, followed by an immune cell response mounted to protect the tissue and promote homeostasis. Then, it is not surprising that these classical anti-inflammatory drugs are effective and prescribed for conditions throughout the body, like the joints (RA, OA), the gut (inflammatory bowel disease), the skin (psoriasis), the lungs (asthma), the blood vessels (vasculitides), or the bones (fractures), which comprise a disparity of mechanisms from trauma injury to autoimmune dysregulation. Furthermore, newer and more sophisticated anti-inflammatory drugs, like biologics, still target the so-called proinflammatory phase, by blocking the actions of various mediators like interleukins or chemokines.^57^ Although the degree of effectiveness may vary across different diseases, depending on which mediators are more prominent in each situation, they are still largely nonspecific for a particular disease or organ system. For example, anti-TNF therapies are effective in RA, colitis and Crohn’s disease, psoriasis and ankylosing spondylitis, or anti-IL-1 antibodies, effective in RA or autoinflammatory disorders. Janus kinase (JAK) inhibitors are also broad-spectrum drugs used to treat RA, colitis, atopic dermatitis, juvenile idiopathic arthritis, or alopecia areata, among other indications.^58^

In line with the integrated view of resolution proposed here, promoting Resolution Pharmacology will also be a universal approach to target inflammatory responses induced by multiple stressors, occurring in multiple system organs. Evidence for this is brought from several distinct studies conducted with animal models of inflammatory diseases, in which proresolving therapies have been tested, as well as from human clinical studies investigating the potential application of these new drugs to diseases with inflammatory component. These will be discussed next.

### B Diseases and conditions susceptible to Resolution Pharmacology

Early indications of the presence of endogenous molecules that actively promote the resolution of inflammation came from studies using simple in vivo research models, like the lipopolysaccharide-induced endotoxemia^59^ suggesting a proresolving role for AnxA1, or the air pouch mouse model to investigate the ability of aspirin-triggered lipoxins and AnxA-derived peptides in downregulating neutrophil responses.^60^ A research model that deserves special consideration, given the vast amount of information and discoveries made on the early days of the resolution field, is the zymosan-induced peritonitis mouse model. This model has helped researchers to (1) Identify new endogenous molecules involved in promoting resolution^61^, ^62^, ^63^; (2) elucidate the cellular mediators of these responses^64^, ^65^, ^66^, ^67^; (3) understand the kinetics of inflammation-resolution mechanisms^68^^,^^69^; (4) test the therapeutic efficacy of proresolving drugs^70^, ^71^, ^72^, ^73^; and (5) establish parameters (ie, resolution indices) that allow the quantification of resolution responses and the evaluation and comparison of proresolving therapeutics.^74^

**Fig. 3:**
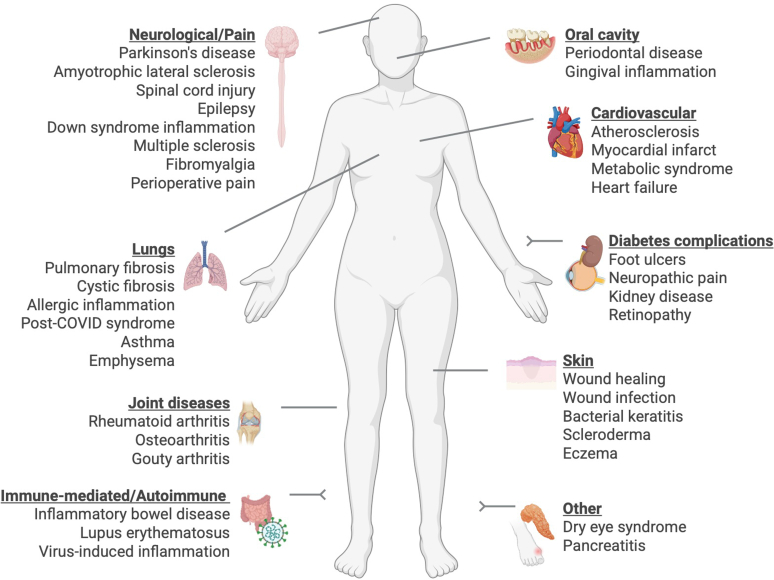
Therapeutic opportunities for Resolution Pharmacology. Proresolving therapies may be used to treat a wide variety of disease indications. Data from preclinical and clinical studies suggest these may include neurological diseases, cardiovascular and lung related diseases, autoimmune conditions, pathologies of the oral cavity and skin, diabetes complications, and others like eye diseases and gout.

However, translational models of disease have been rapidly adopted and applied to the investigation of resolution mechanisms and its therapeutic applications. Without being exhaustive, the health disorders studied as possible indications for proresolving therapeutics include chronic inflammation associated with autoimmune diseases, cardiovascular-related conditions, fibrosis and wound healing, metabolic disorders, microbial inflammation, or conditions associated with neuroinflammation, among many other examples (Fig. 3). Proresolving strategies to resolve arthritis and joint inflammation have been extensively studied. For example, the compound AT-01-KG, a synthetic LXA_4_ mimetic, showed efficacy in a model of gouty inflammation,^75^ whereas various melanocortin-based compounds, including peptides^71^^,^^76^ and small molecules,^72^ have been evaluated in a model of acute joint inflammation, the K/BxN serum transfer murine model, that mimics the acute flares of RA. Other diseases with a major immune component susceptible to proresolving therapeutic strategies include lupus erythematosus,^77^ colitis,^78^ and complications associated with type 1 diabetes like kidney disease,^79^ retinopathy,^80^ or wound healing.^81^ Resolution Pharmacology strategies to treat health disorders of the lungs include the compounds (1) BML-111, which accelerated resolution in a model of ventilator induced lung injury^82^; (2) the FPR2 agonist Quin C1, effective in a model of bleomycin-induced lung fibrosis^83^; (3) RvE1 for pulmonary inflammation^84^; and (4) LxB_4_ for allergic inflammation.^85^ These studies build on the original work of Levy et al^6^ with RvE1 in a model of allergic lung inflammation. Low doses of RvE1 attenuated lung inflammation through reduced levels of IL-23 and IL-6 required to maintain and select Th17 T lymphocytes. Such an effect was associated with higher levels of interferon-gamma.^86^

Cardiovascular and metabolic disorders are also susceptible to proresolving strategies. Examples include atherosclerosis, which improved upon treatment with RvD1,^87^ RvD2,^88^ and LXA_4_,^89^ myocardial infarction using the AnxA1 mimetic RTP-026^90^ and RvE1 to treat sepsis-induced myocardial dysfunction,^91^ as well as metabolic alterations associated with obesity.^92^ A recent review provides a summary of proresolving approaches tested in experimental settings of atherosclerosis and shown to produce significant protection.^93^

Numerous studies in animal models have also elucidated a potential of proresolution therapies for the treatment of a wide range of neurological disorders. These include Parkinson disease,^94^ Alzheimer disease,^95^ amyotrophic lateral sclerosis,^96^ spinal cord injury,^97^ postoperative neuroinflammation,^98^ fibromyalgia,^99^ pain,^100^ and inflammation associated with Down syndrome,^101^ epilepsy,^102^ and multiple sclerosis.^103^

Positive outcomes of proresolving strategies have also been observed in diseases or models of microbial or viral infections, like periodontal disease,^104^ dental pulp regeneration,^105^ gingival inflammation,^106^ skin wound infection,^107^ bacterial keratitis,^108^ post-COVID syndrome,^109^^,^^110^ or cystic fibrosis.^111^

We propose how resolution targets are to a certain extent nonspecific, making Resolution Pharmacology a viable option for therapeutic interventions potentially in multiple inflammatory diseases. As a consequence, each pathological condition may be susceptible to multiple approaches and be treated and controlled by targeting multiple mediators or pathways. An area in which research has been scarce relates to the possibility of using combination therapies to promote resolution. The field has progressed on a *one-mediator-at-a-time* basis, where each major proresolving system (ie, FPR2 agonists, melanocortins, lipid mediators, etc) has been studied and characterized almost in isolation. A recent effort toward the integration of “resolution” knowledge is represented by the launch of the Atlas of Inflammation Resolution (AIR),^112^ a freely accessible web-based resource to study the interplay between multiple cell types, mediators and pathways, and their interactions. Although to date AIR only integrates curated data related to lipid mediators, this platform and its community-sharing approach may expand in the future and include interactions with other types of mediators. Given that the resolution program is a coordinated response, further efforts in this direction may be desirable. Addressing how the combination of mediators targeting different resolution targets may enhance the efficacy of proresolving approaches to treat inflammation represent a therapeutic opportunity which is largely unexplored.

## V Pharmacologically actionable strategies to resolution

There is ample evidence for the modulatory and regulatory properties of resolution mediators and pathways, often generated through knockout mouse colony strategies or using antagonists to specific proresolving receptors, some of which discussed above. Thus, when a given resolution mechanism is inactivated or poorly engaged, experimental acute and chronic inflammatory reactions overshoot and/or are prolonged. In this section, we now move from the abundant preclinical to the limited clinical evidence, to then focus on examples of mediators and receptor targets in relation to proresolving compounds which development is ongoing. To do this, we will describe specific pathways and reason on how our knowledge of their biology is informing the development of new therapeutics to deliver Resolution Pharmacology.

Before we do this, though, a few notes are worthy to be added. The first one is that, against common view, resolution mediators may increase in the circulation or in a given tissue: this could be part of an attempt by our body to avoid overshooting of the host response. Nonetheless, higher levels do not indicate a propathogenic effect. Such a superficial view if often reported when a given proresolving mediator is identified through OMICs approaches, therefore in an unbiased manner. However, this immediate and wrong conclusion is not a novel outcome: for instance, it took around 3 decades to understand that elevated circulating corticosteroid levels in infectious and inflammatory statuses were not provoking disease but, rather, these hormones acted as part of an endogenous protective process to avoid overshooting of the host response, as explained by Munck et al.^113^ In terms of applicability, and in line with what is needed to develop Resolution Pharmacology, even if there is no evidence that a given proresolving mediator or target is absent or reduced in a given pathological condition, this does not exclude the pharmacological benefit that may derive by harnessing the pathway and augmenting its biological effects through mimetics, agonists, or supplementation approaches, as we originally discussed.^1^

The second note is that, in contrast to classical pharmacology, resolution agonists and their relevant receptors do not follow a yin/yang association. Classically, and especially in neuropharmacology, insufficient release of a neurotransmitter associates with an upregulation of its receptor on the postsynaptic elements. Conversely, excessive release of mediator from the presynaptic element causes a downregulation of the receptor on the target neurone. Such an invert relationship may not necessarily apply to the biology of resolution, nor to Resolution Pharmacology. Indeed, there is experimental evidence that both proinflammatory (IL-1*β*) and anti-inflammatory (dexamethasone) molecules induce expression of the proresolving protein, AnxA1.^114^ Therefore, there may be lower expression of a given resolution mediator or even a normal expression of this mediator; nonetheless “pushing” the system with an agonist still can produce the desired pharmacological effect. Such observations may be linked to the inherent controlling mechanisms of proresolving mediators, targets, and pathways.

In fact, it is well known in the field, as reported by several laboratories, that resolution agonists generate bell-shaped concentration or dose responses. The reason behind this shared outcome is still unclear and could be associated to rapid recycling of the receptor, lack of overshooting of the proresolving receptor signaling, and more. In any case, these biological checks underpin the modulatory and regulatory properties of proresolving mediators and the likely “physiological” profile of proresolving drugs. Such inherent cellular checkpoints, while still poorly understood for their molecular mechanisms, would explain the lack of immunosuppressive effects of proresolving therapies, as they will never exceed their effects, or be highly unlikely to do so.

At this point, it is important to discuss briefly the issue of tachyphylaxis or rapid decline of biological efficacy upon repeated drug application. Over the years, this concern has been proposed as a way to exclude engagement in drug discovery programs to obtain receptor agonists. We always found this point of view somewhat surprising, considering the use of alpha-adrenergic agonists as decongestants or mu receptor agonists (methadone) to mitigate the withdrawal effects, over the last decades. It remains, though, an important point to consider, in view of the repeated applications that a proresolving drug will require for chronic diseases. In the previous paragraph we have acknowledged the existence of yet not fully clear cellular mechanisms that regulate drug/receptor interaction to avoid overshooting, hence immunosuppression. In any case, the ultimate verdict will come from experimental data that, as an example, we report later for compounds ACT-389949 and BMS-986235. Our view is that the chemists and pharmacologists engaged in the development of proresolving drugs ought to be aware, not concerned, of this possibility and therefore test it in experimental systems. Rapid recycling of proresolving receptor targets will not lead to tachyphylaxis. Equally, clinical experts that will develop phase I and phase II trials must consider this issue and test it. Ultimately, it may be sufficient to administer the proresolving drug once a day, like the case for resomelagon presented below, and not every 6–8 hours as for ibuprofen or other cyclo-oxygenase inhibitors.

Having covered, albeit succinctly, the development of the concepts of the resolution of inflammation and how it has been molded by a large body of preclinical data, from this point we wish to discuss studies which have quantified resolution mediators in human diseases. However, we must recognize the lack of data in man. In fact, although cytokines—as an example—are measured in virtually every clinical trial through the application of simple multiplex assays, mediators of the resolution of inflammation have long been overlooked and not measured at all. This minimalist approach has greatly hampered the pace of development and translation for this area of science. The diffusion of mass spectrometry analytical protocols or the availability of ELISA for resolution mediators has enabled the more recent acquisition of data from healthy and patient volunteers, together with quantitative analyses in specific phase II trials. In the same vein, unbiased proteomic and genomic analyses have started to reveal presence or absence of proresolving mediators or receptors, thus opening new vistas on human diseases which then could guide on how efficiently—and innovatively—they intervene to mitigate their clinical manifestation. Some of these discoveries and studies will be discussed further.

Next, we provide evidence of human data on a given resolution pathway and then discuss drug development opportunities associated with it, with a clear steer toward clinical trials and analyses in man. We have tried to be comprehensive and attentive to this broad area of research, but we acknowledge that the mediators, targets, and thereof therapeutic developments discussed below are by no mean a fully exhaustive list. Thus, a few relevant trials or discovery programs may have been overlooked, and we apologize in advance to the researchers and investigators who have conducted them. Table 1 reports a few examples on the translational status of proresolving therapies discussed here.

**Table 1:** Examples of proresolving therapies embarked on the translational path Further details can be obtained from clinicaltrial.gov using the NCT number or from published trials using the provided PubMed identification, PMID.

Therapeutic Agent	Proresolving Target/Mechanism	Development Status	Indication
RTP-026	Annexin A1 mimetic FPR2 agonist	Phase Ib/II (NCT06465303)	Acute myocardial infarction
Aclerastide (NorLeu^3^-Ang1-7)	Angiotensin1-7 analog MAS1 receptor agonist	Phase II (PMID: 26029484) Phase III^a^ (NCT01830348; NCT01849965)	Diabetic foot ulcers
Resomelagon (AP1189)	Biased agonist at melanocortin receptors	Phase IIb (NCT06671054) Phase IIa (NCT04456816)	Rheumatoid arthritis Nephrotic syndrome
Dersimelagon (MT-7117)	MC_1_ selective agonist	Phase II (NCT04440592) Phase III (NCT05005975)	Systemic sclerosis Erythropoietic protoporphyria
BVT.115959	Adenosine A2 receptor agonist	Phase Ib/II (NCT00452777)	Diabetic neuropathic pain
15 (R/S)-methyl-LXA_4_	Lipoxin A_4_ derivative FPR2 agonist	Phase II (PMID: 22834636)	Infantile eczema
methyl ester benzo-LXA_4_	Lipoxin A_4_ derivative FPR2 agonist	Phase II (PMID: 34589083)	Periodontal disease
RX-10045	RvE1 analog	Phase II (NCT00799552)	Dry eye syndrome
INBRX-101	Alpha-1-antitrypsin analog	Phase II (NCT05897424)	Emphysema^b^
(R)-Roscovitine	CDK inhibitor/proapoptosis	Phase IIa (PMID: 34961705)	Cystic fibrosis^a^
EV enriched secretome	(multiple)	Phase I/IIa (NCT06545175)	Cochlear implantation^b^

EV, extracellular vesicle.

a Data showed no clinical efficacy.

b Ongoing.

### A Annexin A1 and FPR2 agonists

#### 1 Human biology

The AnxA1/FPR2 pair has resulted as a fundamental pathway in the control of resolution mechanisms, a conclusion based on studies reported by several groups, some of which have been mentioned above. With respect to data in humans, the first studies to indicate alterations in AnxA1 refer to lung pathology. Smith et al^115^ reported degradation of this protein in asthma patient bronchoalveolar lavage fluids,^115^ and absence as well as degradation of AnxA1 is a major feature in nasal epithelial cells from cystic fibrosis patients,^116^ linking defective cystic fibrosis transmembrane conductance regulator (CFTR) to synthesis and/or release of the protein. More recently, a genetic association between a defective *ANXA1* gene and childhood wheezing has emerged.^117^ In settings of infection and inflammation, lower AnxA1 levels have been quantified in the blood of patients affected from severe Dengue disease, the hemorrhagic form,^118^ whereas in Kawasaki disease, substantially lower serum AnxA1 levels were obtained in the patients who present with coronary artery aneurysm, the most severe of this acute and systemic vasculitis.^119^

Moving to cardiovascular diseases, higher cell surface AnxA1 expression has been reported in circulating neutrophils from patients suffering from coronary artery disease^120^: it is unclear whether this is associated with a different disease outcome. Of relevance, a separate study has reported higher AnxA1 on the cell surface of circulating monocytes in response to glucocorticoid treatment: the authors speculate that this cellular response is associated with stable coronary artery disease.^121^ A proteomic study in smooth muscle cells discovered higher AnxA1 levels in human plaque-derived smooth muscle cells from patients without recent acute cerebrovascular symptoms, whereas lower levels were quantified in symptomatic patients; such different levels could modulate cytokine release from these cells, as shown in silencing studies to reduce or abolish intracellular expression of the protein.^122^ The same indication derives from studies with human acute aortic dissection, a life-threatening disease with high morbidity and mortality; herein, analysis of aortic tissue specimens revealed decrease *ANXA1* gene expression in vascular smooth muscle cells presenting an activated (synthetic) phenotype, with higher production of inflammatory mediators and release of matrix metallo-proteases.^123^ Thus, it seems unequivocal that lower AnxA1 protein and gene expression alters the physiological properties of vascular smooth muscle cells toward vascular pathology.

There have been instances where AnxA1 (protein or gene product) was elevated in pathological *versus* physiological settings. We argue above that expression, or higher expression, may not directly indicate a pathogenic role but it could be part of an attempt of our body to correct the insinuating pathology. Such an attempt may be frustrated or in part frustrated, as without the brake potentially afforded by the proresolving mediator, then pathology would be heightened. This is a theoretical analysis as data are scarce. In any case, high levels of AnxA1 protein and mRNA have been detected in the ischemic core of patients with stroke.^124^ In addition, hospitalized patients for acute heart failure with impaired kidney function displayed higher circulating AnxA1 levels and these associated with worse congestion, higher risk for further creatinine elevation, and higher rates of morbidity and mortality.^125^

In fatty liver diseases, AnxA1 protein expression in liver biopsies is inversely correlated with disease severity.^126^ This observation has then prompted preclinical pharmacological studies where delivery of AnxA1 full protein over several weeks attenuated the evolution of nonalcoholic steatohepatitis to fibrosis.^127^ We note a similar pattern in acute pancreatitis. Here, patients with moderate-severe (*n* = 20 patients) and severe disease (*n* = 10 patients) have higher AnxA1 expression in their blood cells compared with control samples (*n* = 32) and samples from patients with mild disease (*n* = 57).^128^ The increase in AnxA1 is predominantly due to its expression in circulating neutrophils. The authors also observed a dynamic regulation with maximal express of AnxA1 mRNA at 24 hours, returning to baseline levels by 168 hours after hospital admission. The “frustrated nature” of this pattern is revealed by the complementary preclinical experiments: here, *AnxA1* knockout mice are more susceptible to pancreas damage in distinct experimental models of disease with higher indices of systemic inflammation. Conversely, delivery of an AnxA1 peptidomimetic afforded tissue protection and regulated the extent of extrapancreatic inflammation.^128^

Initial analyses in a small cohort of patients affected from multiple sclerosis (*n* = 10) revealed remarkably lower AnxA1 plasma levels, with a reduction between 80% and 90% compared with values of the control group: from ∼28 ng/mL in controls to 3–5 ng/mL in patients.^129^

With respect to the receptor target for AnxA1, the formyl peptide receptor 2 or *FPR2*,^130^ a single nucleotide mutation (A>G) in the core promoter has been identified in one individual with history of cardiovascular disease, a gene defect passed to his offspring. Such a mutation reduced *FPR2* promoter activity in vitro and was matched by a 3- to 10-fold in protein and mRNA expression in circulating neutrophils, as compared with cells from their relatives or healthy volunteers expressing the wild-type allele.^131^ Finally, there is evidence for higher FPR2 mRNA in patients suffering from autism, a set of data the authors associated with a potential higher degree of inflammation in these patients.^132^ If the authors are correct in their interpretation, these data suggest an inflammatory component also in this neurological syndrome.

#### 2 Resolution Pharmacology

The translation of this body of research has been slow in coming. Once the N-terminal domain of AnxA1 was identified as the pharmacophore, several peptides spanning different amino acids of this region of the protein have been tested in several in vitro and in vivo assays, the most common being peptide Ac2-26 also referred to as AnxA1_2-26_ or AnxA_1-25_. We have recently reviewed some of the studies conducted with peptide Ac2-26.^133^ However, the only AnxA1 derivative tested in man is RTP-026, a synthetic peptide that spans the longer sequence of the AnxA1 N-terminal domain; it belongs to a group of peptides modified to better resist serine protease mediated proteolysis.^134^ RTP-026 has completed phase I studies successfully and has now entered phase II trial for acute myocardial infarction (NCT06465303; Table 1). An important feature of RTP-026 lies in its potency, much closer to the parent protein than that of peptide Ac2-26, and its selectivity toward human and rodent FPR2: this molecule activates this proresolving receptor at concentration ranging from 10 to 100 nM.^90^^,^^134^

The discovery that the AnxA1 receptor target was FPR2, also the receptor for the proresolving lipid LXA_4_ hence initially termed ALX,^60^ opened the appreciation on the real nature of this G-protein coupled receptor (GPCR). Being phylogenetically close to the chemotactic receptor FPR1, FPR2 was initially considered a proinflammatory receptor. We have discussed above the complexity of inflammation and how in vitro assays of cell activation are unlikely to identify genuine proinflammatory and proresolving properties of a given compound. Equally, we have briefly mentioned the phenotype of mice lacking the ortholog of human *FPR2* gene which again point to a tissue-protective nature of this receptor. There is evidence that ligand-biased signaling may be responsible for the complex effects ascribed to FPR2.^135^^,^^136^

The field of small molecule FPR2 agonists has been quite florid as we reviewed 10 years ago in relation to the patent literature at that time.^1^ This active area of research has been reviewed recently.^137^ Here we focus on the clinical studies conducted with FPR2 agonists. The first successful translation has been achieved by Actelion with the ACT-389949 molecule. This compound was highly selective for human FPR2 and tested in 2 phase I clinical trials in healthy volunteers.^138^ The authors noted how the administration of ACT-389949 was safe and well tolerated in healthy subjects. However, the trial revealed a rapid tachyphylaxis upon repeated administrations, evident through a blunted release of circulating IL-10. Assessment of FPR2 surface receptor levels on monocytes showed a sustained internalization of the receptor targeted by the compound, with loss of regulation of circulating levels of both pro- and anti-inflammatory cytokines (IL-6, IL-8, MCP-1, TNF-α, and IL-10).^138^

A distinct FPR2 agonist was developed by Kyorin Pharmaceuticals and licensed to Bristol Myers Squibb, compound BMS-986235 also identified as LAR-1219. BMS-986235 elicited significant proresolving properties including modulation of proinflammatory cytokine release and polarization of macrophages toward a reparative phenotype. Such pharmacological effects underlined BMS-986235 efficacy in models of myocardial infarction and heart failure with reduced ejection fraction.^139^^,^^140^ Importantly, the same authors provide a convincing molecular explanation for the different pharmacology of ACT-389949 and BMS-986235, by characterizing dynamics of receptor recycling. Thus, although ACT-389949 addition to FPR2 expressing cells provides rapid and prolonged receptor internalization, with also receptor catabolism through the ubiquitination pathway, BMS-986235 addition caused a rapid time-dependent receptor internalization, so that FPR2 re-appeared on the cell surface after 60–90 minutes.^141^ Such a difference was due to the engagement of a different *β*-arrestin postreceptor activation with either compound. BMS-986235 has been tested in phase I trials for safety in healthy volunteers (NCT03335553) with anticipated staging for phase II trials in heart attack patients.

Two further points are as follows. First, drug discovery programs based on the structure of LXA_4_—which also activates FPR2—have also been conducted and will be discussed below. Here we cite a recent review that provides an excellent account of lipoxin-based drug discovery approaches.^142^ Second, the large datasets available for FPR2 agonists in preclinical settings may open drug development pathways aside those discussed above or currently under investigation, eg, hypertension^143^ or heart failure associated with autoimmune diseases.^144^

### B Angiotensin 1-7 and the MAS1 receptor

#### 1 Human biology

The renin-angiotensin system (RAS) is a central pathway widely known for its role in blood pressure regulation. More recently, the protective and regenerative effects of RAS activation have been appreciated through activation of the injury upregulated MAS1 proto-oncogene, a GPCR belonging to the class A orphan family. Circulating angiotensin I, a 10-amino-acid peptide (Ang1–10), can be cleaved by angiotensin-converting enzymes (ACEs) to smaller peptide sequences. Using a refined analytical protocol, it has been possible to quantify these peptides in the blood of 11 health volunteers and 3 patients under kidney dialysis. The authors detected Ang1–10 and Ang1–8 at concentrations between 1 and 10 pM in healthy subjects, whereas Ang1–9, Ang1–7, and Ang1–5 could not be detected in any of the 14 samples.^145^ ACE2 converts angiotensin II (Ang1–8) to Ang1–7, a functional antagonist of Ang1–8, with vasodilatory, antiproliferative, antiangiogenic, and anti-inflammatory properties. An open-label clinical investigation following intravenous administration of human recombinant ACE2 demonstrated the causal increase of peptides including Ang1–7, Ang1–8, and Ang1–5, quantified by liquid chromatography-tandem mass spectrometry.^146^

It is evident that detection in blood may only be partially relevant, as tissue-protective molecules ought to be produced and be present at the site of injury. Moreover, several studies have emphasized the positive effects produced through inhibition of the *detrimental tissue RAS* pathway or obtained following activation of the *protective tissue RAS pathway*: such beneficial outcomes have been shown in settings of cardiovascular, inflammatory, and autoimmune disease. Relevant to this discussion is the protective tissue RAS pathway that is centered on the angiotensin II type 2 receptor plus ACE2 to yield Ang1–7 which then activates MAS1. The starting mediator remains angiotensin II, a vasoconstrictor that can exhort pathological mechanisms when it stimulates pathological pathway or exerts protective effects when stimulating the angiotensin II type 2 receptor. The original studies by da Silveira and colleagues reported the anti-inflammatory and antiarthritic effects of Ang1–7.^147^^,^^148^ Subsequently, the same researchers demonstrated the genuine proresolving pharmacology of the heptapeptide, through the promotion of polymorphonuclear cell apoptosis and macrophage efferocytosis of apoptotic cells in arthritis and asthma, respectively.^149^^,^^150^ Many of these biological properties were reliant on the activation of MAS1 receptor, expressed on both murine and rat cells and tissues, as well as on human neutrophils and eosinophils. The pharmacological properties of the Ang1–7/MAS1 pathway have been recently reviewed.^151^ Altogether this wealth of work provides the scientific background for developing analogs to Ang1–7 or small molecular agonists to MAS1.

There is pharmacological potential against bone diseases since Ang1–7 stimulate matrix synthesis with concomitant reduction in bone-resorptive cytokines, altogether yielding a higher osteoprotegerin-to-RANK ligand ratio.^152^ A translation of these observation occurs in the context of periodontal disease, where human gingival samples contain higher levels of components of the protective tissue RAS pathway as compared with inflamed gingival samples.^153^^,^^154^ Preclinical studies have convincingly indicated beneficial effects of Ang1–7 in models of infectious diseases,^52^^,^^53^ where there is ability to modulate the host response to underline the synergistic effect with antibiotics.

#### 2 Resolution Pharmacology

Aclerastide, or NorLeu^3^-Angiotensin1–7, produces tissue-protective effects by accelerating the healing process, as shown by the improvement of tissue healing in a range of preclinical models. In a phase II clinical trial with 77 patients, aclerastide significantly accelerated healing of diabetic foot ulcers (reduced the median time for healing to 8.5 weeks from 22 weeks in placebo). These beneficial effects have been ascribed secondary to augmented (1) progenitor cell proliferation, (2) vascularization, (3) collagen deposition, and (4) re-epithelialization^155^ (Table 1). However, these effects have not been confirmed in a subsequent phase III trial conducted in diabetic patients affected from plantar ulcers (NCT01830348; NCT01849965). Mechanistically, this failure in the larger study may be due to unwanted effects of the compound through the release of active oxygen species and matrix metalloproteinase, as shown with mechanistic studies in experimental diabetic wounds.^156^ It remains to be demonstrated whether these effects are off-target effects or also occurring downstream MAS1 activation. In any case, although more studies are required, the availability of a safe MAS1 agonist like aclerastide can enable effective translation of this body of work, perhaps for clinical indications other than wound healing; examples could be, to remain with topical application, skin pathologies and lung infectious diseases.

Other therapeutic approaches to exploit the RAS system, mainly the protective tissue RAS axis, have been recently reviewed.^151^ Worth recalling here an oral formulation of Ang1–7 (which is encapsulated into hydroxypropyl *β*-cyclodextrin) that has been tested in a randomized clinical trial: this treatment showed promising effects against muscle injury postexercise in humans, through reduction in blood levels of TNF-*⍺* and an analgesic effect.^154^ Ongoing work on this tissue-protective pathway is represented by the peptidomimetic CGEN856S (from Compugen), a MAS1 agonist that protects against experimental infarction^157^ and the nonpeptide agonist AVE0991, as recently summarized.^158^ A search on clinicaltrial.gov did not reveal ongoing clinical investigations with these 2 molecules, though at this juncture one cannot exclude testing in phase I trials, which in some cases do not need to be registered.

### C Melanocortin receptor agonists

#### 1 Human biology

Melanocortin receptors form a family of 5 GPCRs with remarkably similar homology but equally remarkably distinct functional roles.^159^ MC_1_ in melanocytes controls skin pigmentation, whereas MC_3_ and MC_4_ in the brain regulate appetite. Activation of MC_2_ on the adrenal glands induces the release of cortisol, whereas MC_5_, found in exocrine glands, regulates sebum production. In fact, each of the 5 melanocortin receptors (MCRs) presents value as a therapeutic target, and preclinical and clinical research efforts are currently ongoing for all receptors for different indications.^160^ These receptors are stimulated by the endogenous melanocortin peptide agonists (ACTH, *⍺*-, *β*-, and *γ*-melanocyte stimulating hormone [MSH]), with the exception of MC_2_, only activated by ACTH. Of relevance for Resolution Pharmacology, receptors MC_1_ and MC_3_ seem to be more prominent because of their expression in immune cells and other relevant cell types like fibroblasts. Reported proresolving actions of melanocortins include increased efferocytosis and acceleration of resolution in mouse peritonitis.^71^^,^^72^ A long-standing limitation affecting advancement in the field is the lack of truly selective antibodies for the experimental detection of these receptors in cells and tissues, a problem affecting GPCR research in general. Another limitation that also prevented scientists obtaining a better definition of MCRs as targets is the general nonselective profile of agonists, both endogenous and synthetic. It is then possible that other MCRs like MC_4_ and MC_5_ may also present value as proresolving targets, if their expression can be reliably confirmed in relevant cells or tissues. Indeed, the advancement of MC_1_ as a proresolution target was likely driven by the development of truly MC_1_-selective compounds, like BMS-470539.^161^ Research from multiple labs using this compound has provided robust preclinical target validation evidence for MC_1_ for the treatment of joint inflammation,^162^ neuroinflammation,^163^ diabetic retinopathy,^164^ cartilage degradation,^165^ lung injury,^166^ or nephropathy.^167^

All melanocortin receptors are coupled to Gs proteins, hence signaling canonically through cAMP, although several other pathways have been described including phosphorylation of extracellular signal-regulated kinase and others like elevations in intracellular Ca^2+^, p38 phosphorylation, or JAK/STAT pathway.^160^ In terms of regulation and how this system is modulated in physiology and pathology, evidence from literature points toward a regulation at the ligand level rather than at the receptor level.

Numerous reports suggest altered levels of the endogenous melanocortin peptide *⍺*MSH in different inflammatory conditions. Elevated levels of *⍺*MSH were found in plasma of HIV-infected patients, whereas reduced levels were observed in septic syndrome patients at onset, with levels returning to normal after recovery.^168^ Altered levels of circulating *⍺*MSH were also detected in association with obesity,^169^ chronic fatigue syndrome,^170^ or older adults frailty.^171^ Importantly, *⍺*MSH levels may also have predictive value as a biomarker in several situations. An observational study suggested that higher *⍺*MSH levels in ischemic stroke patients may predict long-term outcome,^172^ suggesting that an early intervention with this peptide may have therapeutic benefit. Similarly, it was suggested that circulating levels of this peptide could predict cardiac event occurrence in patients with coronary artery disease.^173^ Another report suggests that in patients with osteonecrosis of the femoral head, systemic levels of *⍺*MSH are negatively associated with radiographic progression and symptoms severity.^170^ In RA, however, levels of *⍺*MSH were increased locally in the synovial fluid, rather than systemically, suggesting a local production at inflammation site.^174^ This report, published in 1994, was already pointing to the fact that the levels of an “anti-cytokine” molecule and proinflammatory mediators at sites of inflammation may display a positive correlation.

Although melanocortin peptide production seems to be promptly modulated under pathological conditions, there is no clear evidence of modulation of the receptor expression at the cellular level, and when these increases are observed in tissues, they most likely suggest infiltration of immune cells which carry the receptor with them, rather than true transcriptional modulation. Then, to harness Resolution Pharmacology, the presence of the target, not necessarily its modulation, may be sufficient to trigger a response and promote resolution.

#### 2 Resolution Pharmacology

Different strategies have been followed over the years to develop new ligands to target melanocortin receptors, leading to the development of peptide analogs, small molecules, and other innovative strategies including nano-delivery systems,^167^ antibody-based melanocortin agonism,^175^ targeted radiotherapies utilizing MC_1_ agonism to direct the drug to the target cancer cell^176^ (NCT05496686) or theranostics precision delivery technologies using MC_1_ agonism-based imaging to guide radiotherapy^177^ (NCT05655312).

At variance from other classes of proresolving drugs, it is noteworthy that several melanocortin drugs and formulations have reached marketing authorization. Therefore, they represent the first in class within the Resolution Pharmacology arena. These include a synthetic shorter version of ACTH peptide, referred to in literature as ACTH_1-24_, cosyntropin or tetracosactide, used mostly for adrenal function diagnostic purposes but this compound may also be prescribed for inflammatory conditions such as gout, inflammatory bowel disease, or RA. Another example relevant for inflammatory conditions is Clinuvel’s afamelanotide (Scenesse), consisting of an *⍺*MSH stable peptide analog, [Nle^4^,D-Phe^7^]-*⍺*MSH approved for the treatment of erythropoietic protoporphyria, a form of skin phototoxicity associated with severe inflammation and pain. Although the therapeutic efficacy derives from the induction of melanogenesis in the skin, the anti-inflammatory and antioxidative actions of this peptide are also likely to play an important role in providing therapeutic benefit.^178^

Other peptide analogs currently at clinical testing stage include PL9643^179^ and PL8177,^76^^,^^180^ under development by Palatin Technologies for the treatment of ocular diseases and ulcerative colitis, respectively. The first of these peptides, PL9643, recently completed a phase III study on 575 patients (MELODY-1, NCT05201170) reporting rapid and complete symptoms resolution in patients with dry eye disease. A synthetic porcine version of ACTH (SNP-ACTH(1-39) Gel), developed by Cerium Pharmaceuticals, is currently on phase III trial for the treatment of primary membranous nephropathy, an autoimmune disease of the kidneys causing inflammation and damage to the glomeruli filtration function in which MC_1_ agonism has shown promise.^181^ This drug also received orphan drug designation by the Food and Drug Administration.

Regarding small molecules, a potent and selective MC_1_ agonist may hold promise for the treatment of fibrotic diseases.^55^ This orally available molecule, dersimelagon or MT-7117, developed by Mitsubishi Tanabe Pharma, recently completed a phase II study in subjects with diffuse cutaneous systemic sclerosis (NCT04440592) although results on efficacy have not been reported to date.

The GPCR nature of melanocortin receptors also offers new therapeutic modalities beyond classical agonism/antagonism, and strategies exploiting allosteric modulation and biased signaling have already been explored for these receptors.^182^ Confo Therapeutics have developed a platform technology aiming to develop new molecules to modulate GPCRs activity in various ways like agonism, inverse agonism, or allosteric modulation. They currently have an MC_1_ agonist for the treatment of immune-mediated and fibrotic diseases at early stages of development. Another example of alternative ways of GPCR modulation is offered by the small molecule resomelagon (previously referred to as AP1189),^72^ an orally administered biased agonist under clinical development by SynAct Pharma for the treatment of nephrotic syndrome (NCT04456816), virus-induced hyperinflammation (RESOVIR-2 study, NCT06917001), and RA (phase IIb, ADVANCE study, NCT06671054). For the latter indication, reported results from completed trials indicate that resomelagon achieved significant response over placebo particularly in a subpopulation of patients who presented elevated C-reactive protein levels at baseline, without causing immunosuppression, a key selling point of Resolution Pharmacology.

The melanocortin system is the only GPCR family for which endogenous antagonists have been identified, termed agouti signaling protein and agouti-related neuropeptide, which also present inverse and biased agonistic activity.^182^ This suggests that alternative modes of therapeutic interventions could be designed, in addition to direct receptor agonism, by for instance inhibiting or blocking the antagonist. Such a strategy has been explored by vTv Therapeutics, which tested the compound TTP435, an agouti-related protein inhibitor, for the management of obesity (NCT00779519). We identify the need for a better understanding of the role of MCR antagonists during inflammatory responses and this may lead to similar strategies to harness Resolution Pharmacology.

### D Adenosine and the A_2A_ receptor

#### 1 Human biology

Adenosine is an autacoid produced at specific tissue sites through the metabolism of ATP and AMP which are dephosphorylated by the action of adenosine kinase and another hydrolase. Once formed, adenosine is released into the extracellular space and can be taken up by other cells. Similarly, many cells including neutrophils and endothelial cells express ectonucleoside like CD39 and ecto-5′-nucleotidase-like CD73 which can lead to synthesis of adenosine.^183^ The whole system is complex, and it may be concerning that circulating levels adenosine can be quite even in healthy individuals (around 1 *μ*M); they can increase up to 4–10 *μ*M in settings of sepsis.^184^ However, in analogy with what discussed for other proresolving mediators, the most relevant value remains the concentration of adenosine within the tissue site: to this end, a concentration of less than 1 *μ*M has been detected in normal tissues, and this can increase up to 100 *μ*M in settings of inflammation and ischemia: for example, these concentrations have been quantified in synovial fluids of patients with RA.^185^ Such high concentrations, although obviously correct, may detract somewhat from the physiopathological relevance or impact that adenosine may have, especially in comparison with other proresolving mediators that are active at low nanomolar range.

Another element of complexity lies in the fact that 4 adenosine receptors exist, and they may produce distinct pharmacological effects. Relevant to our discussion is mainly the A_2A_ receptor, from the seminal study of Montesinos et al^186^ showing the role of this receptor in wound healing to several other studies that have demonstrated the genuine regulatory effects of adenosine through this receptor.^187^ However, “receptor interference” may take place and for instance adenosine can increase the release of neutrophil-extracellular traps through activation of the A_1_ receptor, whereas agonism at the A_2A_ receptor would inhibit this release.^188^ This potential receptor-mediated yin-yang has been called upon for an interesting mechanistic proposal, whereby high tissue levels of adenosine could desensitize A_1_ receptor signaling, pushing the pendulum toward A_2A_ receptor signaling with downstream inhibition of cell activation.^189^ Aside modulation of neutrophil reactivity through the A_2A_ receptor, this pathway is also operative during macrophage efferocytosis, another cardinal sign of resolution. Herein, release of adenosine by the efferocytotic macrophage leads to autocrine/paracrine A_2A_ receptor activation to dampen the release of proinflammatory chemokines, avoiding propagation of the inflammatory response and ensuring that the process promotes resolution.^49^ A further modulatory process takes place during macrophage phagocytosis and efferocytosis with upregulation of the A_2A_ receptor and downregulation of the proinflammatory receptors of this autacoid.^190^ Such receptor modulation follows similar regulation upon activation of the monocytic cell line (THP-1 cells) with proinflammatory cytokines, with increased A_2A_ receptor expression.^191^ This is reminiscent with what reported above for FPR2, and the overall concept discussed in earlier paragraphs on the functional association between inflammatory and resolution phases of the inflammatory response.

Altogether, it seems to us that—albeit with specific differences in given districts, like asthma—the A_2A_ receptor is the most druggable proresolving receptor of adenosine. This proposal is corroborated by a study of 700 subjects where identification of 4 single-nucleotide polymorphisms of *ADORA2A* (rs2236625, rs2236624, rs4822489, and rs5751876) has been detected. Polymorphism rs4822489 was associated with the severity of chronic heart failure.^192^ Although interesting to some degree, the authors conclude that larger cohorts ought to be examined before reaching a firm causal association.

#### 2 Resolution Pharmacology

An important pharmacological application of adenosine biology is its augmentation posttreatment with methotrexate, which is discussed below. Equally, though less relevant here, is the use of 8-chloro-adenosine, that belongs to a class of chemotherapy drugs called purine analogs that work to treat cancer by their ability to interfere with cancer cell metabolism, causing cell death: it has been tested for chronic lymphocytic leukemia (NCT00714103). Trials have been conducted with adenosine in cardiovascular settings, including its application through preconditioning to afford myocardial protection during surgery repair of congenital heart defects (NCT00881686). A more recent trial has tested whether addition of adenosine to standard intermittent warm blood cardioplegia in patients undergoing minimally invasive operations (mitral valve surgery) reduces markers of heart injury like cardiac troponin (NCT02681913). Indeed, too many trials come up when one enters the keyword “adenosine” in clinicaltrial.gov: important to focus on some of them relevant to this review though virtually all of them do not post the obtained results. In any case, NCT00298636 by Xsira Pharmaceuticals tested the effect of treatment with adenosine in perioperative pain. More recently, another A_2A_ agonist, BVT.115959, has been tested for efficacy and tolerability in patients affected from diabetic neuropathic pain (NCT00452777) (Table 1).

In line with the pharmacological opportunity to promote resolution mechanisms by *pushing or activating* specific endogenous mediators and targets of tissue repair and protection, methotrexate (15 mg/wk orally over a 12-week period) has been shown to increase adenosine levels in 10 patients suffering from inflammatory arthritis (NCT00184886). Later we will resume, succinctly, similar strategies based on the nutraceutical supplementation with omega-3 fatty acids.

CGS-21680 is a highly selective A_2A_ receptor agonist. It must be a useful pharmacological tool, as we could not find studies where this compound had been tested in healthy people or in patients. For instance, tested on synovial cells from patients with OA and RA, CGS-21680 inhibited the release of IL-6 only in RA cells, however at the same time increased levels of TNF-*⍺* and IL-10.^193^ Similarly, CGS-21680 exerts proangiogenic effects in human dermal endothelial cells, an effect at least in part linked to the mobilization of AnxA2, involved in plasminogen activation.^194^

More interesting is the testing of the highly selective A_2A_ receptor agonist binodenoson against cardiac ischemia, a study funded by Pfizer (NCT00944970; NCT00944294), side by side against adenosine itself. Although results of these trials have not been published, a study with binodenoson in patients affected from asthma has been published: 87 healthy young adult volunteers with documented mild, intermittent asthma were recruited to test again safety rather than efficacy. The endpoint was that this selective A_2A_ agonist was safe also in the presence of mild asthma.^195^

Another A_2a_ receptor agonist is regadenoson (Lexiscan), able to vasodilate the coronary arteries: NCT00859833 assessed a dose-response investigation with respect to coronary hyperemia. This was followed by a subsequent registered trial (NCT01809743). Both regadenoson and binodenoson add value compared with adenosine in cardiac physiology testing, since they have a better pharmacokinetics. A side-by-side comparison has been made, and the authors have concluded that regadenoson has shorter duration of action than binodenoson, whole still delivering a comparable hyperemic response: this represents an advantage for better control in clinical application, avoiding unwanted changes in systemic blood pressure.^196^ Indeed, the phase I trial with binodenoson in healthy subjects has been published, together with its full pharmacokinetics characteristics.^197^

We speculate that there may be an active Biotech (eg, Purine Pharmaceuticals) and BigPharma (Pfizer mentioned above) engagement in the development of adenosine receptor-based therapeutics; however, we could not find much more at this juncture.

### E Lipid mediators and their receptors

#### 1 Omega-3 and Omega-6 derived molecules

##### a Human biology

Lipid mediators represent a major arm of the biology of resolution and, conversely, a great potential for Resolution Pharmacology. Original concepts of endogenous anti-inflammation focused on LXA_4_ and LXB_4_, both derived from arachidonic acid,^198^ lipid mediators have become cardinal to the science of resolution since the 2007 review cited above.^2^ The observation that LXA_4_ could actually promote efferocytosis^199^ helped to crystallize the fundamental principles of resolution. Subsequently, the discovery of novel classes of proresolving lipid mediators that derive from omega-3 fatty acids like eicosapentaenoic acid (EPA), docosahexaenoic acid (DHA), and docosapentaenoic acid (DPA) by Serhan propelled this field of research, so several studies have described expression and function of these molecules, some mentioned above. Here we go over some exemplars to highlight outcomes relevant to the scope of this review.

In patients affected from RA, a seminal study reported an association between specific lipid mediators and responsiveness to treatment.^200^ Patients who responded to synthetic disease-modifying antirheumatic drugs have higher circulating levels RvD4 and maresin 1 (MaR1) than patients resistant to therapy (association between specific lipid mediators and responsiveness of RA patients to treatment). Assessment of circulating lipid mediator concentrations 6 months after treatment initiation establishes that differences between responders and nonresponders are maintained, with a decrease in concentrations (RvD4, 15R-LXA_4_, and n-3 docosapentaenoic acid-derived MaR1 in patients resistant to disease-modifying antirheumatic drugs therapy). Systemic analyses have been complemented by quantification of lipid mediators in the synovial tissue or arthritic joint. Barden et al^201^ studied 36 patients undergoing arthrocentesis and a same number of healthy volunteers, all taking 2.4 g/d of omega-3 supplementation for 4 weeks. In synovial fluids, the authors detected molecules derived from EPA and DHA, including the precursors 18-HEPE and 17-HDHA, and unveiled a negative association between synovial quantities of RvE2 and the pain score recorded by these patients. A subsequent groundbreaking study presented deep analyses of synovial tissue cells and mediators from patients with RA at different disease states. It was found that a proresolving MerTK+ macrophage subtype is more abundant in the synovia of patients in remission, and that this cell type releases higher levels of RvD.^202^ This is probably the first observation in humans to demonstrate appearance of proresolving macrophage during inflammation resolution, together with the preferential production of a proresolving mediator. One way to gain mechanistic cues in arthritis is to make comparison between RA (which is always a florid inflammatory condition) and OA (which is often not an inflammatory condition). Thus, analyses of the synovial fluids of 18 patients with RA and 26 patients with OA revealed higher concentrations of proinflammatory lipid mediators (eg, prostaglandins and leukotrienes) as well as proresolving lipid mediators (including lipoxins, resolvins, and protectin D1) in the former group of samples.^203^ In agreement, another study with OA synovial tissue and synovial fluid cells reported a lower activity of 5-LOX and 15-LOX in OA compared with RA, with no apparent differences in COX-derived products.^204^

Broadening autoimmune diseases to pathologies other than RA, there is a recent study in amyloid lateral sclerosis with the observation that low levels of circulating resolvins associate with faster disease progression.^96^ Focusing on their receptors, both GPR32 (receptor for RvD1, RvD1_n-3 DPA_, and LXA_4_) and GPR18 (receptor for RvD2) are expressed to a lower extent in blood mononuclear cells. The authors propose the existence of a proresolving lipid mediator-directed neuroprotective response that could inform different therapeutic strategies and the application of novel biomarkers for amyloid lateral sclerosis.^96^ Multiple sclerosis is another disruptive neurological pathology. Here, overlapping results to those observed in amyloid lateral sclerosis have been reported. Thus, an impaired expression of enzymes and receptors associated with several proresolving lipid mediators has been reported in blood-derived leukocytes of patients affected from multiple sclerosis. Addition of mediators detected at a lower level (namely, LXA_4_, LXB_4_, RvD1, and protectin D1) to monocytes from these patients reduced their activation and the ensuing production of proinflammatory cytokines.^205^ The authors offer a similar conclusion, that specific proresolving lipid mediators could be of value in diagnosis as well as in promoting the development of new therapies for multiple sclerosis.

Within inflammatory diseases, a clinical trial determined expression of LXA_4_ as well as AnxA1 in gingival crevicular fluid of patients suffering from grade 3 periodontitis (NCT06700161); these analyses would have been compared with levels of a main proinflammatory cytokine IL-1*β*. At present, we think the results obtained with this trial have not been published. A published report from the same team has described cytokine and chemokine profiles, as measured by multiplex technology, which positively associated with periodontal disease severity.^206^ In settings of asthma, patients affected from severe disease have ∼90% lower levels of LXA_4_ in their bronchoalveolar lavage fluid as compared with samples from patients presenting a milder form of the disease. This reduction in the proresolving lipid mediator is matched by a lower degree of expression of both the synthesizing enzyme (15-LOX) and the receptor FPR2.^207^ In children affected from cystic fibrosis, an imbalance between LXA_4_ and leukotriene B_4_ has been demonstrated, an observation the authors have associated to the nonresolving chronic inflammation that typifies this disease.^208^ The 2 studies provide the backbone to the pharmacological application of LXA_4_ or its analogs as discussed below. In the context of gut inflammation, and specifically colon cancer that emerges from unresolved inflammation, a recent study reports deep analysis of lipid mediators. Mass spectrometry analysis of *n* = 40 samples from patients and healthy controls revealed a biased toward proinflammatory molecules (eg, leukotrienes and prostaglandins) in colon tumors *versus* normal matched mucosa.^209^ The conclusion was presence of an inflammatory milieu within the tumor microenvironment that sustains the cancer due to an upregulation of biosynthetic enzymes required for the synthesis of proinflammatory lipid mediators. Conversely, expression of enzymes that synthesize proresolving lipid mediators was low in patients, causing a remarkable reduction or absence of specific molecules. Altogether this study is another recent one that breaks the ice by quantifying proresolving molecules in diseased human samples. At the same time, it confirms the importance of deep analyses of the affected tissue as a way to unveil pathogenic mechanisms.^209^

Several clinical studies have analyzed the effect of omega-3 supplementation in cardiovascular diseases, too many to be reported here, with more recent studies that have focused on the proresolving lipid mediators themselves. A prospective observational study has investigated the plasma levels of lipid mediators in patients with ST-elevation myocardial infarction (*n* = 15) for a week after the event. Measurements were made at 3 timepoints, in comparison with patients with stable coronary artery disease (*n* = 10) and healthy controls (*n* = 10). A temporal regulation of DPA- and DHA-derived molecules emerged, with high levels early during ST-elevation myocardial infarction.^210^ Moreover, an interesting shift from proinflammatory LTB_4_ to T-series resolvins was also observed in this investigational study. In settings of peripheral artery disease, which is due to the process of atherosclerosis, 5-day oral administration of an enriched marine oil supplement was assessed in healthy volunteers and patients with peripheral artery disease (*n* =10 in each group). This subacute treatment with omega-3 produced an elevation of many proresolving lipid mediator families, together with a higher ratio against prostaglandin levels.^211^ These measurements and associated changes were married with higher phagocytosis of circulating monocytes and neutrophils. In addition, lower CD18 and CCR2 expression was observed on classical monocytes.

In settings of infection, like meningitis associated with tuberculosis, a phase II trial where aspirin was administered on top of corticosteroid therapy, changes in lipid mediators in the cerebrospinal fluid were observed. In particular, a reduction in proinflammatory thromboxane A_2_ occurred in a dose-related fashion to aspirin dose, and this was complemented by an increase in proresolving protectins.^212^ In the same pathological settings of meningitis with tuberculosis, a study tried to relate lipid mediator levels to 80-day survival.^213^ Cumulative amounts of proresolving mediators—including DHA-derived MaR1 and EPA-derived E-series resolvins—in cerebrospinal fluids are negatively related to disease severity. Moreover, survivors displayed higher levels of 5-LO-derived 13-series resolvin (RvT)2, RvT4, and 15-epi-LXB_4_, compared with those who died. When patients responded to high-dose aspirin, this treatment reduced (1) thromboxane A_2_, (2) brain infarcts, and (3) mortality and concomitantly increased protectin levels in the cerebral spinal fluid.^213^

As indicated, here we are presenting a small number of studies conducted with either omega-3 supplementation or with drugs known to augment proresolving lipid mediators like aspirin, to provide examples on how these treatments are linked to these families of molecules. Other reviews have described the biology of proresolving lipid mediators and their receptors.^93^^,^^214^^,^^215^ In relation to the latter, it is interesting to cite a recent study which identified a frameshift mutation in one of the isoforms of leucine-rich repeat-containing GPCR 6 (LGR6), the receptor for MaR1.^216^ Subjects homozygous for this variant had significantly lower levels of LGR6 on selected white blood cells. Moreover, lack of LGR6 impacted on neutrophil and monocyte phagocytosis of applied bacteria and their ability to respond to the cognate ligand MaR1. Finally, interrogation of the largest database that UK Biobank offers, presence of the polymorphism rs74355478 in the *LGR6* gene was associated with a higher incidence of viral infections.^216^

##### b Resolution Pharmacology

A search through registries for clinical trials (eg, clinicaltrial.gov) allows to identify dozens of studies where lipid mediator levels, including those for resolvins and lipoxins, are quantified and tested either as diagnostic biomarkers to predict specific disease manifestations (eg, see NCT03095157 for postthoracic pain) or as companion diagnostic biomarkers to predict efficacy of a given drug or medicine (some examples have been discussed above, like the methotrexate study in patients with RA). Not many trials are registered where actual new composition matters or analogs to lipid mediators are tested for clinical efficacy. This is in face of burgeoning research efforts in the inflammation resolution area and, within it, the large number of discovery science and preclinical studies with proresolving lipid mediators. Few examples of drug discovery efforts, however, exist and some are selected below. We observe that LXA_4_ and RvE1 have, so far, been the focus of translational studies as well as been used as scaffolds to design and characterize novel synthetic analogs. Of great interest is the annotation that a first-in-class proresolving biologic has also emerged from this line of research.

The first lipoxin analog was produced by Berlex,^217^^,^^218^ and since then, several efforts have been made to characterize new chemical entities by modifying the structure of LXA_4_, to increase its pharmacokinetics, and possibly also pharmacodynamic, properties including stability and oral bioavailability.^219^ However, in terms of human pharmacology, the first in man study has used the analog 15(R/S)-methyl-LXA_4_ which was tested against infantile eczema in a randomized controlled trial.^220^ Sixty patients with infantile acute or subacute facial eczema were enrolled, with an age range between 1 and 12 months. Topical application of the compound over a 3-week period afforded significant improvement of the clinical manifestation, with significant efficacy as early as after 3 applications, and maximal effect at day 10 posttreatment: 15(R/S)-methyl-LXA_4_ had similar efficacy as the reference treatment (the corticosteroid mometasone) and, of importance, no safety concerns were raised at all^220^ (Table 1). A study in asthma conducted with 50 children, tested 5(S),6(R)-LXA_4_ methyl ester and BML-111 (a useful compound that acts as lipoxin mimetic): both treatments resulted to be safe and afforded an amelioration of the pulmonary parameters under analysis.^221^

A larger study has been conducted in 127 patients affected from periodontal disease. The LXA_4_ analog methyl ester-benzo-lipoxin A_4_ was applied daily through oral rinse over a 28-day period; safety as well as gingival inflammation and periodontal pocket depths were assessed regularly. The treatment resulted in significant reduction in gingival inflammation and bleeding, assessed through a specific score system.^106^ Oral treatment with the proresolving compound elicited systemic effects as emerged from the quantification of serum levels of proresolving and proinflammatory lipid mediators, with higher levels of the former (including MaR1, MaR2, LXA_4_, and LXB_4_) over placebo or no rinse application, and lower levels of the latter molecules (LTB_4_, PGE_2_, and PGD_2_) (Table 1).

Attenuate Therapeutics have been established to characterize LXA_4_ analogs synthesized and developed preclinically at University College Dublin,^73^^,^^142^ with compounds AT-01-KG and AT-02-CT probably being the most advance in development. These analogs have been designed to resist oxidation and metabolic degradation, supporting an application for oral delivery.

Resolvyx was established over 10 years ago to harness the proresolving properties of resolvins, mainly RvE1. Indeed, RX-10001, which is the commercial name for the natural RvE1 structure, has been tested in a phase I study in healthy volunteers (NCT00941018). The RvE1 analog, termed RX-10045 (Table 1), has been tested in preclinical models of eye disease in the rabbit^222^^,^^223^ prior to a phase II trial designed to assess safety and efficacy in patients with dry eye syndrome (the trial is registered as NCT00799552); results have been positive and reported in a press-release form.^224^ A newly opened trial (NCT06356844) aims to add resolvins to epidural steroid injection and assess the potential beneficial effects for patients suffering from inflammatory low back pain; it is unclear which resolvin or resolvin-like molecule will be used here, as oral administration is stated in the trial narrative.

Thetis Pharma are developing a platform to discover RvE1 analogs, unclear to which development status at present. The analog TP-317 is being developed as an antagonist to the LTB_4_ receptor BLT_1_, as well as a chemerin receptor 1 (Chemerin_1_, also known as ChemR23) receptor agonist. Within this space, Ose Immunotherapeutics have developed an activating monoclonal antibody to Chemerin_1_, the GPCR that conveys the pharmacological activities of RvE1.^225^ Interestingly, this antibody termed OSE-230 activates the receptor in a proresolving like manner: this means that once applied onto Chemerin_1_-expressing cells, it replicates the proresolving signaling evoked by RvE1, and not the proinflammatory signaling that occurs after application of chemerin.^226^ This first-in-class proresolving biologics has been acquired by AbbVie in a partnership agreement with OSE Immunotherapeutics, to be clinically developed for treatment of chronic inflammatory diseases. Optimistically, we should hear about progress of this molecule over the next few of years.

Thetis are also testing in phase Ia combination between metformin + EPA. This combination therapy for oral administration will be compared with metformin administered together with icosapent ethyl in healthy volunteers (NCT02113163). This trial brings us to acknowledge the large number of clinical investigations of omega-3 fatty acid supplementation in patients suffering from several disease types, initially the focus was on inflammatory and cardiovascular diseases, more recently investigations covering also metabolic and central nervous system diseases. These studies are too many to cover here and partially out of scope, as our focus remains Resolution Pharmacology. Nonetheless, it is accepted that nutraceutical approaches are of therapeutic relevance and could well be taken as cotherapy and/or coadjuvant together with classical medicines.^227^ There are systematic reviews that offer a scholar view of these studies—especially those where randomized control trials were applied—with beneficial effects in RA^228^^,^^229^ as well as cardiovascular^230^ and muscle^231^ pathologies. Alongside this approach is the development of softgels enriched with polyunsaturated fatty acids by Solutex; the clue here the presence of standardized levels of the EPA and DHA together with that of the lipid mediator precursors 17-hydroxy-DHA and 18-hydroxy-EPA. NCT03434236 will test Lipinova in patients undergoing orthopedic surgery.

#### 2 Cannabinoids

Endocannabinoids like anandamide and 2-arachidonoyl-*sn*-glycerol clearly fall under the classification of lipid mediators, as they are derivatives of arachidonic acid, like LXA_4_ and LXB_4_. There are a few studies on cannabinoids and inflammation, with protective effects mainly associated to activation of the cannabinoid receptor type 2 and with a focus on gastrointestinal inflammation.^232^ However, it is scarcely determined whether cannabinoids are endowed with genuine proresolving properties as those described above for lipid and nonlipid mediators. Their pharmacology has predominantly been studied in the context of neurological pathologies (eg,^233^, ^234^, ^235^ as well as the control of pain), which clearly is associated to acute and chronic inflammatory situations. There are a couple of interesting “intersections” between the cannabinoid system and the proresolving lipid mediators under discussion here. In 2012, a report identified LXA_4_ as a positive allosteric modulatory to the cannabinoid receptor type 1, CB_1_.^236^ This molecular interaction was reflected in a potentiation by LXA_4_ of the protective effects elicited by a CB_1_ ligand against loss of spatial memory provoked by a *β*-amyloid fragment: this finding fits with the protective effects of cannabinoids against Alzheimer disease. More recently, an excellent study conducted with cannabidiol demonstrated the ability of this compound to provoke a class switch in the synthesis of lipid mediators by myeloid cells. The authors show that cannabidiol reduced production of leukotrienes through the 5-LOX while promoted synthesis of proresolving lipid mediators by stimulation of phospholipase A_2_-mediated release of polyunsaturated fatty acids and activation of 15-LOX.^237^ This switch in biosynthesis also occurred in experimental settings of acute inflammation, though was not translated into an overt anti-inflammatory effect of cannabidiol. A search on cannabidiol in clinicaltrial.gov reveals over 150 entries; however, the vast majority of these trials relate to neurological, psychiatric, and pain control conditions. Out of the few which may be relevant to this discussion, NCT01037322 aimed to determine the potential efficacy of cannabidiol in inflammatory bowel disease, whereas NCT04607603 and NCT04911127 aimed to determine efficacy against OA of the knee and RA, respectively. As in the case for several of these registered trials, results have not been posted.

##### a Human biology and pharmacology

With the awareness that cannabinoids may be identified yet as proresolving lipid mediators, in the context of this discussion on Resolution Pharmacology we wish to cite the development of fatty acid amide hydrolase (FAAH) inhibitors. We have presented the approach of potentiating a given pathways by supplementing with the substrate (omega-3 fatty acid supplementation); herein FAAH inhibitors represent an example of how to potentiate endogenous pathways by inhibiting catabolism of specific mediators. A recent review illustrates this approach and provides an update on drug development programs based on FAAH inhibitor, like JNJ-1661010 and PF-04457845, as well as other strategies specific for the endocannabinoid system.^238^ The therapeutic potential focus remains diseases of the central nervous system.

#### 3 Sphingosine 1-phospate

Under the umbrella of translational approaches that harness the biology of specific lipid mediators, on top of arachidonate- and omega-3-derived molecules, there is also a line of research centered on sphingosine 1-phospate (S1P). S1P is present in the circulation produced by red blood cells as well as the endothelium; this mediator can bind to 5 distinct GPCRs, of which the S1P receptor 1 (S1P_1_) is the most relevant for therapeutic exploitation.

##### a Human biology and pharmacology

In systemic lupus erythematous, S1P levels have been reported to be elevated in serum samples of juvenile patients (*N* = 15),^239^ whereas a larger study with over 100 patients revealed the ratio ceramide:S1P as the best discriminator marker between patients and healthy controls.^240^

The existence of S1P-based therapeutics has been recently reviewed,^241^ with the description of 4 Food and Drug Administration-approved ligands to S1P receptors for autoimmune diseases: fingolimob, ozanimod, siponimob, and ponesimod. These agonists have therapeutic use as functional antagonists, as they cause marked postbinding receptor downregulation. Such a mechanism risks to bring unwanted secondary effects on the vasculature.^241^ In lupus patients, an exploratory investigation with amiselimod showed the S1PR1 modulator to reduce anti-double-stranded DNA levels in 9 of 17 people, with 7 of 17 people displaying a reduction in disease activity.^242^ New approaches to use the biology of S1P for therapeutic purposes are under development, including the use of chaperone-bound S1P like ApoM-S1P, a complex with therapeutic potential in ocular diseases.^243^

Similarly to the comment above for cannabinoids, it is yet unclear whether S1P is a genuine proresolving molecule in the way we have classified other mediators, lipid, or protein; nonetheless, the fact that S1P agonists are under development not to act as functional antagonists but to elicit direct tissue-protective effects makes the whole area of research interesting and potentially relevant to Resolution Pharmacology.

### F Other strategies in the translational landscape

#### 1 Alpha-1-antitrypsin

The acute phase response represents a primordial reaction against insults and dangers that precedes the development of antibodies and of the adaptive immune response, as scholarly reviewed in reference.^244^ There are hundreds of proteins synthesized and release as part of this reaction, the large majority from the liver. Textbooks refer to acute phase proteins (APPs) as important sensors of infection and tissue damage, yet their main translational value lies as diagnostics for systemic inflammation, the top exemplar here being C-reactive protein, ubiquitously measured in clinical trials as an improbable or insufficient marker of systemic inflammation. The hepatic synthesis of APPs in mainly governed by 2 pluripotent cytokines like IL-1 and IL-6, the same cytokines that also activate the hypothalamus/pituitary axis leading to the release of hormones like ACTH and the downstream synthesis of glucocorticoids (discussed above in the section on "Melanocortin receptor agonists"). As glucocorticoids exert a permissive effect on the hepatocyte,^245^^,^^246^ optimizing their response to IL-1 and IL-6, a coordinated and integrated multiorgan response to injury and infection by our body emerges. The “buffering” role of APP—meant to capture in the circulation cytokines as well as exogenous factors—has been accepted for some time though evidence begun to come to light for potential different properties and functions. First, macrophages and endothelial cells can synthesize and release APP, raising the option that these proteins may exert effects locally, at site of inflammation perhaps. Second, some of the APP can act directly on target cells to incite or enhance reparative processes. We focus here on alpha-1-antitrypsin (AAT), a serine protease inhibitor which physiopathological role has been ascribed to inhibition of elastase and similar proteases like proteinase 3 and cathepsin G.

##### a Human biology

Genetic deficiency of AAT, fortunately quite rare, deregulates the homeostatic relationship between proteases and antiproteases, with absence of inhibition on neutrophil elastase and consequent breakdown of elastin within the pulmonary interstitium. This circuit then promotes lung inflammation which may conduct to fibrosis and lung emphysema. Patients with AAT deficiency (AATD) would vary in the degree of AAT reduction, and this impacts disease severity.^247^ Patients with AATD are treated with supplementation of plasma-derived AAT: a randomized controlled phase II trial demonstrated that augmentation therapy with AAT 60 mg/kg per week over 24 months in patients with AATD (92 receiving the augmentation therapy and 85 treated with placebo) delayed onset and progression of emphysema.^248^

##### b Resolution Pharmacology

The fact that AAT is clinically used may represent an opportunity for other diseases, through a rapid repurposing of the drug. We discovered an abundance of AAT in experimental resolving exudates^249^ and observed its ability to protect against joint inflammation in experimental setting of inflammatory arthritis and OA.^250^ There may be a potential for translation as based on these 2 observations. First, a study with 31 patients suffering from OA reported a significant reduction in circulating AAT as compared with values measured in 29 age-matched control individuals.^251^ Second, another clinical investigation observed lower levels of AAT in the cartilage from patients with OA.^252^^,^^253^ Analysis of synovial fluids from OA shoulder joints has revealed lower levels of AAT in early with further reduction in the liquid biopsy obtained from patients with late phase OA of this joint.^254^

Mechanistically, AAT main pharmacological effect is consequent to inhibition of serine proteases. Nonetheless, other mechanisms that could underpin its anti-inflammatory and, potentially, proresolving bioactions have been reported. Thus, macrophages can internalize AAT which then acts on the glucocorticoid receptor to exert anti-inflammatory and antimycobacterial effects, through attenuation of cytokine synthesis and increased killing of mycobacteria.^255^ The authors followed this study with an assessment of gene regulation in a macrophage cell line by AAT in a glucocorticoid-receptor-mediated fashion.^256^ The functional interaction with glucocorticoids is of interest, since these hormones have also been associated to the discovery of AnxA1. A second crosstalk could link AAT and AnxA1 more directly. The 37 kDa AnxA1 protein can be cleaved by serine proteases like elastase and proteinase-3, and this is a catabolic phenomenon that removes the main active domain of the protein (the N-terminal region^257^). Therefore, synthetic and natural inhibitors of protease increase AnxA1 levels by preventing its cleavage in settings of neutrophilic inflammation: in this manner, these molecules exert anti-inflammatory effects independent from a direct inhibition of their main targets, the serine proteases.^258^ The same mechanistic association has been shown for plasmin and the prevention of AnxA1 degradation has been associated with an impact on macrophage polarization.^259^ We would not be surprised if future studies will demonstrate a pharmacological interaction between AAT and AnxA1, at least in inflammatory settings with high presence of myeloid cells.

There may be a potential for AAT use to mitigate diabetes complications. Thus, a study with 240 patient samples found lower AAT levels (∼15-fold decrease) in patients with diabetic retinopathy.^260^ In terms of intervention, a phase II randomized control clinical trial (NCT02005848) observed that AAT delivery to patients with early onset type 1 diabetes (adolescents) afforded encouraging protection of pancreatic cells.^261^ This medicine is produced by Kamada biopharmaceutical company.

INBRX-101 is a human recombinant protein with 2 moieties of AAT fused to one Fc domain. As such, this derivative is more stable and should have better pharmacokinetics, especially if associated with prolonged treatment of patients affected from chronic disease, like AATD and more. A phase I trial (NCT03815396) seems to indicate good safety, with 2 registered phase II trials for replenishment therapy and emphysema prevention in patients with AATD (NCT05897424; NCT05856331) (Table 1). Recently, INBRX-101 has been purchased by Sanofi,^262^ making us to foresee that there may be further development in this area of pharmacology, for AATD and potentially for other pathologies as discussed herein.

#### 2 Gelsolin

Another plasma protein of current interest is gelsolin, not a classical APP but rather an actin-binding protein highly expressed in muscles. There are 3 isoforms of gelsolin, of which plasma gelsolin is the extracellular isoform,^263^ shown to decrease in settings of acute injury and inflammation. A study in 78 patients suffering from RA revealed lower plasma gelsolin levels as compared with 62 healthy control people.^264^ This was accompanied by presence of gelsolin/actin complexes in the synovial fluids of these patients. The authors suggested that gelsolin may be exerting local and possibly systemic anti-inflammatory effects. Our own studies identified gelsolin to be abundant in resolving exudates^249^ and, similarly to AAT discussed above, reported its ability to protect against joint inflammation.^250^ Gelsolin knockout mice displayed higher inflammation to urate crystals and in endotoxemia.^265^

An investigation on gelsolin expression patterns in human synovia revealed different levels among healthy, RA, or OA tissues. Both macrophage-like synoviocytes and fibroblast-like synoviocytes expressed gelsolin and the same was true for chondrocytes: in patients with OA, the authors quantified a significant reduction in synovial fluid gelsolin levels.^266^ Clearly, these initial observations need to be mitigated by the number of patient samples tested (which ranged from 6 to 8) and also by the fact that different stages of the respective disease may determine if not regulate the amount of gelsolin being released in the synovial fluids. BioAegis Therapeutics Inc have run a phase Ib/IIa study on safety and efficacy of human recombinant plasma gelsolin in patients effected from commonly acquired pneumonia (NCT03466073), together with a study in COVID patients (NCT04358406). A more recent trial, which is actively recruiting, is going to test human recombinant plasma gelsolin in patients suffering from acute respiratory distress syndrome (NCT05947955).

These 2 examples of proteins endowed with tissue-protective actions downstream cellular phenotype switches indicate the potential that whole protein therapies may have in a variety of difficult-to-treat diseases. We reason that plasma proteins and specifically APPs may be endowed with pharmacological activities that are more complex than currently appreciated, which often is restricted to their buffer-like properties. Another example is that of lactoferrin: 2 tripeptides derived from this protein (phenylalanine-lysine-aspartic acid and phenylalanine-lysine-glutamic acid or FKD and FKE, respectively) are able to switch the phenotype of human macrophages toward a reparative/resolving one.^267^ Interestingly, Ariel et al identified naturally occurring peptides (eg, FKECHLA) that contain the active sequence but were less potent.^267^

#### 3 Targeting immunometabolism

Over the last 2 decades, thanks to the groundbreaking studies of O’Neill et al,^268^ there has been a burgeoning interest in metabolic changes that take place in inflammatory and stromal cells in settings of inflammatory diseases. Borrowing from the cancer field and the Warburg effect, inflammatory cells change their source of ATP upon activation, from oxidative phosphorylation to glycolysis. Thus, M1-like macrophages make use of glucose hence have higher glycolysis, whereas M2-like macrophages are reliant on oxidative phosphorylation.^268^ The same dichotomy applies to T helper cells, with T helper cells and regulatory T cells using glycolysis or oxidative phosphorylation as energy source. Since the shift in polarization from M2-like to an M1-like macrophage is a prominent proresolving mechanism (that can be extended to shift from active to resting or reparative phenotypes in many other cell types), compounds that can promote this switch through changes in immunometabolism could well be part of the current review. We are not going to cover all bases here and list the multiple approaches one can take to alter cell metabolism as a way to temper cell activation in inflammation. A recent review provides a good summary to this purpose, with an interesting interconnection with therapies we have discussed here, like glucocorticoids.^269^

Itaconate is a by-product of the Kreb’s cycle which reprograms mitochondrial metabolism: in macrophages it underpins the effect of glucocorticoids.^270^ Elevating itaconate levels by favoring its biosynthesis or delivery of itaconate itself or mimetics could be viable strategies to reprogram macrophage activity. To this end, 4-octyl itaconate has been used in several preclinical settings. An intriguing observation has been recently reported, whereby addition of 4-octyl itaconate to mouse macrophages promotes secretion of AnxA1, offering a further link to the resolution mediators and mechanisms discussed herein.^271^ The same effect is obtained after addition of dimethyl fumarate. Fumarate and itaconate induce a modification of cysteine residues in signaling proteins and transcription factors involved in inflammation (see reference^269^ for a review).

In terms of clinical trials, the itaconate derivative termed SIT-011 or LY3839840 has been tested in healthy volunteers. The trial (NCT06153355) with no results posted on clinicaltrials.gov. It remains to be seen if it will be published, probably after completion of a phase IIa trial. Dimethyl fumarate is a cell permeable derivative of fumarate which in been tested in patients who are taking methotrexate for RA alongside a combination therapy design (NCT00810836).

We are confident that the next decade will see a further explosion of pharmacological approaches that aim to harness cell metabolism to impact on the immune status of a given organ or tissue. These might be further translation of itaconate or fumarate, as well as approaches that regulate glucose uptake through inhibition of specific glucose transporters (eg, see reference^272^ or targeting lactic acid accumulation^273^).

### G Further therapeutic opportunities in the resolution space

We conclude this overview on the state of the art in the Resolution Pharmacology landscape by citing other opportunities that are underdevelopment or could be developed in the near future. From a clinical perspective, therapies that promote repair and regeneration, if based on the fundamental biology of resolution mediators, pathways, and mechanisms, are genuinely examples of Resolution Pharmacology.

Fundamental processes that impact the phenotype of immune cells as well as of stromal cells are those of senescence and apoptosis. Thus, for long time we have known the opportunity to promote apoptosis of neutrophils to resolve inflammation, especially if this process is complemented by efficient efferocytosis. Indeed, induction of apoptosis and promotion of efferocytosis are cardinal signs of resolution biology. Without repeating studies and data presented above, we focus here only on potential Resolution Pharmacology therapies founded on these approaches.

The original work of Rossi et al^274^ identified inhibitors of cyclin-dependent kinase (CDK) as genuine proresolving molecules by promoting apoptosis of neutrophils and eosinophils. As such, this approach would accelerate resolution of granulocytic inflammation by pushing programmed cell death with consequent removal of apoptotic cells by resident macrophages. (R)-roscovitine (seliciclib or CYC202) is the compound tested here with subsequent studies identified CDK7 and CDK9 as targetable enzyme to elicit this pharmacology.^275^ NCT02649751 tested (R)-roscovitine in patients suffering from cystic fibrosis, bearing the most common F508del *CFTR* mutation (Table 1). This disease is characterized by waves of lung infection and inflammation, the latter characterized by marked neutrophil recruitment. The data of this phase II randomized control trial have been published: although roscovitine (administered at 200 mg, 400 mg, and 800 mg) was safe and well tolerated, no evident clinical efficacy emerged.^276^ As only 34 patients were recruited to the trial, it is clear that multiple factors including pharmacokinetics of the drug and doses used may have had impacted on the outcome. Moreover, aside clinical parameters and safety recording, there was no assessment of the treatment on neutrophil numbers and extent of apoptosis, making unclear whether roscovitine exerted its primary pharmacological action. Another CKD inhibitor is the molecule AT7519 which is being tested in patients affected from metastatic tumors (eg, NCT02503709). Based on the wealth of preclinical data that sustain the beneficial effects of promoting neutrophil apoptosis in several disease settings, it is plausible that future therapies harnessing this biology might demonstrate clinical efficacy. At the same time, it is equally possible that a proapoptotic effect ought to be part of a broader pharmacodynamics profile (ie, in addition to other bioactions like proefferocytosis or switch in cell phenotype) in order to yield clinical efficacy. This second hypothesis will vouch against highly selective proapoptotic proresolving drugs. An interesting application of this biology is represented by the use of apoptotic cells themselves as a therapy which has been proposed, and discussed, for patients with RA.^277^ This proposal builds on a phase I/IIa clinical trial where a single infusion of apoptotic cells ameliorated the clinical signs of graft-versus-host disease.^278^ We note registration of a clinical trial where apoptotic cell infusion will be tested in patients with RA, though the status is currently unknown (NCT02903212).

The seminal studies by Alivernini et al^202^ have identified a synovial macrophage which is reparative and characterized by the proefferocytotic receptor MerTK. We have discussed above how the process of efferocytosis represents a fundamental mechanism in resolution biology with strong evidence in several preclinical settings including that of atherosclerosis.^279^^,^^280^ CD47 acts as an antiphagocytic receptor and anti-CD47 therapies have been tested in patients with cancer, using the humanized anti-CD47 antibody termed magrolimab. This treatment was effective in reducing tumor volume in patients with relapsed or refractory lymphoma. A recent retrospective analysis of a small cohort of these patients, bearing vascular disease including arterial calcification, revealed a reduction in vascular inflammation and plaque volume. These data, albeit indirectly, indicate that by removing an efferocytosis blocker hence, presumably promoting intraplaque efferocytosis, can be effective to reduce or control the disease.^281^ These preliminary data offer a proof of concept on a proresolution therapy in atherosclerosis and demand larger and more directly designed trials. Other evidence emerging in this field have identified a protein called developmental endothelial locus-1 as a genuine proresolving factor that promotes efferocytosis.^282^^,^^283^

The combination of studies and trials in myocardial infarction and heart failure together with these initial observations in atherosclerosis makes us reason that cardiovascular pathologies may be one of the top diseases to be successfully targeted by Resolution Pharmacology.^284^ In addition to cardiovascular diseases, we have provided an update on proresolving therapies been developed for the treatment of RA. In the same space other therapeutic approaches have shown efficacy in experimental medicine studies of RA and other joint diseases, as previously reviewed.^285^ The concept put forward here of tissue-specific mechanisms to resolve disease, or amenable to intervention and resolve disease, is important and reminds us of the “local” features of the inflammatory reaction. IL-9 promotes type 2 innate lymphoid cells to expand regulatory T cells and promote resolution of joint arthritis: patients with RA in remission display higher numbers of IL-9 positive cells in the joint.^286^ In a recent study, we provide preclinical evidence that distinct immune mechanisms may operate in the joint and the heart of the same experimental animal in settings of inflammatory arthritis: proresolving therapy impacted on these mechanisms in a tissue-specific manner.^144^

We have touched upon example of drug repositioning or how a given drug can become the starting point for drug discovery programs. An example here is the one of lidocaine, which chemical structure has been modified to reduce anesthetic properties while retaining or augmenting its ability to modulate the host response.^287^ Compounds JMF2-1, JM25-1, and JME-173 have been tested and demonstrated activity with an ever-decreasing effect on neuronal transmission.^288^ It remains to be determined the developmental path for these molecules, including whether they are able to promote genuine proresolving properties while modulating the host response.

Finally, an opportunity inherent Resolution Pharmacology lies in the capacity of proresolving therapies to effect multiple actions as discussed previously, and, as such, ensure the sharp turning of the inflammatory reaction to enable resolution. It is possible that such a complex and integrated effect may also be achieved through *polypharmacology* approaches. Clearly, this is not the review where mesenchymal stem cell therapy should be discussed both for the divergent topic and the elevated number of studies performed with this cellular therapy. However, although it is unlikely that mesenchymal stem cell therapy is effective by transformation into the required target cell (eg, cardiomyocyte or chondrocyte), still this therapeutic treatment may elicit tissue repair and regeneration. The latter is the desired outcome of Resolution Pharmacology.

As an example of this multipronged poly-pharma approach, we report an open-label phase I/IIa trial to investigate the safety of intracochlear application of a secretome from mesenchymal stem cells enriched in extracellular vesicles. Trial NCT06545175 has recently started with ongoing recruitment of patients undergoing cochlear implantation (Table 1). There is evidence that extracellular vesicles, with their ample portfolio of proteins, lipids, and nucleic acids (microRNA), can promote a series of downstream actions leading to, or at least facilitating, resolution of inflammation followed by tissue repair and regeneration.^289^^,^^290^

MED’INN’Pharma is developing a secretome (termed SuperMApo) from efferocytotic macrophages to mitigate inflammation while promoting resolution in settings of gut, joint, and brain inflammation.^291^^,^^292^ It is plausible that SuperMApo also contain extracellular vesicles, as these microstructures have been demonstrated to have functional roles in efferocytosis and macrophage reprogramming.^293^

## VI Conclusions and future perspectives

Ten years from 2015 when we proposed Resolution Pharmacology as a novel and unified area of pharmacology, progresses have been substantial. Hundreds of studies have been conducted in the preclinical arena and the biology of resolution is now fully endorsed by the scientific community. In the same vein, therapeutic development programs based on this biology have started: here we attempted to present them in a broad way, covering distinct families of mediators and targets used for the design of potential new drugs. Thus, the proposal that the resolution of inflammation can guide the development of novel therapies is breaking through, yet it is clear we are still away from full exploitation of the concepts and science of resolution. Apart from few examples like the MC mimetics approved by the European, American, and Australian regulatory agencies, several other molecules are still in transition while other proresolving drugs have been stopped in their development, even if these interruptions may have been based on financial or strategic decisions. We remain optimistic and exhort the engagement of the pharmaceutical industry to complement and propel forward the groundbreaking work currently done mainly by academics and biotech companies. The drug discovery knowledge and power of the pharmaceutical industry is required to get us over the finishing line. Such a hope is determined only by the conviction that proresolving drugs may be of great benefit to patients by (1) limiting or controlling their pathology and/or (2) taming down comorbidities or secondary organ damage to, ultimately, improve their quality of life.

We remain convinced that Resolution Pharmacology, with its multipronged efficacy and moderate modulation of inflammatory processes, can deliver its benefits: our prediction is that by 10 years from now we would have the answer. Only time will tell whether we are correct.

## Conflict of interest

MP declares to be a shareholder of ResoTher Pharma ApS and a director of William Harvey Research Limited; is an advisory board member for SynAct Pharma AB; and is involved in the following commercial projects: SynAct Pharma AB, ResoTherPharma, TXP Pharma AG, BioAegis and MRX Medical. He is an inventor on a patent related to AnxA1 proresolving peptides (European Patent 3533457 B1). TMM declares to be a director of William Harvey Research Limited and has a consulting role and collaborative project with SynAct Pharma AB.

## References

[1] Perretti M., Leroy X., Bland E.J., Montero-Melendez T. (2015). Resolution pharmacology: opportunities for therapeutic innovation in inflammation. Trends Pharmacol Sci.

[2] Serhan C.N., Brain S.D., Buckley C.D. (2007). Resolution of inflammation: state of the art, definitions and terms. FASEB J.

[3] Hench P.S., Kendall E.C., Slocumb C.H., Polley H.E. (1949). The effects of the adrenal cortical hormone 17-hydroxy-11-dehydrocorticosterone (Compound E) on the acute phase of rheumatic fever; preliminary report. Proc Staff Meet Mayo Clin.

[4] Flower R.J. (2003). The development of COX2 inhibitors. Nat Rev Drug Discov.

[5] McInnes I.B., Schett G. (2017). Pathogenetic insights from the treatment of rheumatoid arthritis. Lancet.

[6] Levy B.D., Clish C.B., Schmidt B., Gronert K., Serhan C.N. (2001). Lipid mediator class switching during acute inflammation: signals in resolution. Nat Immunol.

[7] Gobbetti T., Coldewey S.M., Chen J. (2014). Nonredundant protective properties of FPR2/ALX in polymicrobial murine sepsis. Proc Natl Acad Sci U S A.

[8] de Paula-Silva M., da Rocha G.H.O., Broering M.F. (2021). Formyl peptide receptors and annexin A1: complementary mechanisms to infliximab in murine experimental colitis and Crohn’s disease. Front Immunol.

[9] Babbin B.A., Laukoetter M.G., Nava P. (2008). Annexin A1 regulates intestinal mucosal injury, inflammation, and repair. J Immunol.

[10] Damazo A.S., Yona S., D’Acquisto F., Flower R.J., Oliani S.M., Perretti M. (2005). Critical protective role for annexin 1 gene expression in the endotoxemic murine microcirculation. Am J Pathol.

[11] Schloer S., Hübel N., Masemann D. (2019). The annexin A1/FPR2 signaling axis expands alveolar macrophages, limits viral replication, and attenuates pathogenesis in the murine influenza A virus infection model. FASEB J.

[12] Kraft J.D., Blomgran R., Bergström I. (2022). Lipoxins modulate neutrophil oxidative burst, integrin expression and lymphatic transmigration differentially in human health and atherosclerosis. FASEB J.

[13] McArthur S., Gobbetti T., Kusters D.H.M., Reutelingsperger C.P., Flower R.J., Perretti M. (2015). Definition of a novel pathway centered on lysophosphatidic acid to recruit monocytes during the resolution phase of tissue inflammation. J Immunol.

[14] Serhan C.N., Savill J. (2005). Resolution of inflammation: the beginning programs the end. Nat Immunol.

[15] Medzhitov R. (2008). Origin and physiological roles of inflammation. Nature.

[16] Dahlman I., Kaaman M., Olsson T. (2005). A unique role of monocyte chemoattractant protein 1 among chemokines in adipose tissue of obese subjects. J Clin Endocrinol Metab.

[17] Weisberg S.P., Hunter D., Huber R. (2006). CCR2 modulates inflammatory and metabolic effects of high-fat feeding. J Clin Invest.

[18] Mazurek T., Zhang L., Zalewski A. (2003). Human epicardial adipose tissue is a source of inflammatory mediators. Circulation.

[19] Vyas V., Sandhar B., Keane J.M. (2024). Tissue-resident memory T cells in epicardial adipose tissue comprise transcriptionally distinct subsets that are modulated in atrial fibrillation. Nat CardioVasc Res.

[20] Medzhitov R. (2021). The spectrum of inflammatory responses. Science.

[21] Buckley C.D., Filer A., Haworth O., Parsonage G., Salmon M. (2004). Defining a role for fibroblasts in the persistence of chronic inflammatory joint disease. Ann Rheum Dis.

[22] Bottini N., Firestein G.S. (2013). Duality of fibroblast-like synoviocytes in RA: passive responders and imprinted aggressors. Nat Rev Rheumatol.

[23] Khodeneva N., Sugimoto M.A., Davan-Wetton C.S.A., Montero-Melendez T. (2022). Melanocortin therapies to resolve fibroblast-mediated diseases. Front Immunol.

[24] Sodin-Semrl S., Taddeo B., Tseng D., Varga J., Fiore S. (2000). Lipoxin A4 inhibits IL-1 beta-induced IL-6, IL-8, and matrix metalloproteinase-3 production in human synovial fibroblasts and enhances synthesis of tissue inhibitors of metalloproteinases. J Immunol.

[25] Sun W., Ma J., Zhao H. (2020). Resolvin D1 suppresses pannus formation via decreasing connective tissue growth factor caused by upregulation of miRNA-146a-5p in rheumatoid arthritis. Arthritis Res Ther.

[26] Hannon R., Croxtall J.D., Getting S.J. (2003). Aberrant inflammation and resistance to glucocorticoids in Annexin 1^−/−^ Mouse. FASEB J.

[27] Damazo A.S., Yona S., Flower R.J., Perretti M., Oliani S.M. (2006). Spatial and temporal profiles for anti-inflammatory gene expression in leukocytes during a resolving model of peritonitis. J Immunol.

[28] Lima K.M., Vago J.P., Caux T.R. (2017). The resolution of acute inflammation induced by cyclic AMP is dependent on annexin A1. J Biol Chem.

[29] Galvão I., Vago J.P., Barroso L.C. (2017). Annexin A1 promotes timely resolution of inflammation in murine gout. Eur J Immunol.

[30] Dufton N., Hannon R., Brancaleone V. (2010). Anti-inflammatory role of the murine formyl-peptide receptor 2: ligand-specific effects on leukocyte responses and experimental inflammation. J Immunol.

[31] Krönke G., Katzenbeisser J., Uderhardt S. (2009). 12/15-lipoxygenase counteracts inflammation and tissue damage in arthritis. J Immunol.

[32] Habouri L., El Mansouri F.E., Ouhaddi Y. (2017). Deletion of 12/15-lipoxygenase accelerates the development of aging-associated and instability-induced osteoarthritis. Osteoarthr Cartil.

[33] Arnardottir H.H., Dalli J., Norling L.V., Colas R.A., Perretti M., Serhan C.N. (2016). Resolvin D3 is dysregulated in arthritis and reduces arthritic inflammation. J Immunol.

[34] Fredman G., MacNamara K.C. (2021). Atherosclerosis is a major human killer and non-resolving inflammation is a prime suspect. Cardiovasc Res.

[35] Fredman G., Hellmann J., Proto J.D. (2016). An imbalance between specialized pro-resolving lipid mediators and pro-inflammatory leukotrienes promotes instability of atherosclerotic plaques. Nat Commun.

[36] Kojima Y., Volkmer J.P., McKenna K. (2016). CD47-blocking antibodies restore phagocytosis and prevent atherosclerosis. Nature.

[37] Thorp E., Cui D., Schrijvers D.M., Kuriakose G., Tabas I. (2008). Mertk receptor mutation reduces efferocytosis efficiency and promotes apoptotic cell accumulation and plaque necrosis in atherosclerotic lesions of apoe−/− mice. Arterioscler Thromb Vasc Biol.

[38] Li Y., Gerbod-Giannone M.C., Seitz H. (2006). Cholesterol-induced apoptotic macrophages elicit an inflammatory response in phagocytes, which is partially attenuated by the Mer receptor. J Biol Chem.

[39] Davan-Wetton C.S.A., Pessolano E., Perretti M., Montero-Melendez T. (2021). Senescence under appraisal: hopes and challenges revisited. Cell Mol Life Sci.

[40] Lipscomb M., Salfate Del Rio I., Eid M. (2025). Resolvin D2 limits senescent cell accumulation in atherosclerotic plaques. Vascul Pharmacol.

[41] Ovadya Y., Landsberger T., Leins H. (2018). Impaired immune surveillance accelerates accumulation of senescent cells and aging. Nat Commun.

[42] Younes R., LeBlanc C.A., Hiram R. (2022). Evidence of failed resolution mechanisms in arrhythmogenic inflammation, fibrosis and right heart disease. Biomolecules.

[43] Hiram R., Xiong F., Naud P. (2024). An inflammation resolution-promoting intervention prevents atrial fibrillation caused by left ventricular dysfunction. Cardiovasc Res.

[44] Helgadottir A., Manolescu A., Thorleifsson G. (2004). The gene encoding 5-lipoxygenase activating protein confers risk of myocardial infarction and stroke. Nat Genet.

[45] Leoni G., Patel H.B., Sampaio A.L.F. (2008). Inflamed phenotype of the mesenteric microcirculation of melanocortin type 3 receptor-null mice after ischemia-reperfusion. FASEB J.

[46] Halade G.V., Kain V., Hossain S., Parcha V., Limdi N.A., Arora P. (2022). Arachidonate 5-lipoxygenase is essential for biosynthesis of specialized pro-resolving mediators and cardiac repair in heart failure. Am J Physiol Heart Circ Physiol.

[47] Kain V., Liu F., Kozlovskaya V. (2017). Resolution agonist 15-epi-lipoxin A_4_ programs early activation of resolving phase in post-myocardial infarction healing. Sci Rep.

[48] Tourki B., Kain V., Pullen A.B. (2020). Lack of resolution sensor drives age-related cardiometabolic and cardiorenal defects and impedes inflammation-resolution in heart failure. Mol Metab.

[49] Köröskényi K., Duró E., Pallai A. (2011). Involvement of adenosine A2a receptors in engulfment-dependent apoptotic cell suppression of inflammation. J Immunol.

[50] Maaser C., Kannengiesser K., Specht C. (2006). Crucial role of the melanocortin receptor MC1R in experimental colitis. Gut.

[51] Leoni G., Alam A., Neumann P.A. (2013). Annexin A1, formyl peptide receptor, and NOX1 orchestrate epithelial repair. J Clin Invest.

[52] Zaidan I., Tavares L.P., Sugimoto M.A. (2022). Angiotensin-(1-7)/MasR axis promotes migration of monocytes/macrophages with a regulatory phenotype to perform phagocytosis and efferocytosis. JCI Insight.

[53] Zaidan I., Carvalho A.F.S., Grossi L.C. (2024). The angiotensin-(1-7)/MasR axis improves pneumonia caused by Pseudomonas aeruginosa: extending the therapeutic window for antibiotic therapy. FASEB J.

[54] Tavares L.P., Melo E.M., Sousa L.P., Teixeira M.M. (2022). Pro-resolving therapies as potential adjunct treatment for infectious diseases: evidence from studies with annexin A1 and angiotensin-(1-7). Semin Immunol.

[55] Kondo M., Suzuki T., Kawano Y. (2022). Dersimelagon, a novel oral melanocortin 1 receptor agonist, demonstrates disease-modifying effects in preclinical models of systemic sclerosis. Arthritis Res Ther.

[56] McInnes I.B., Gravallese E.M. (2021). Immune-mediated inflammatory disease therapeutics: past, present and future. Nat Rev Immunol.

[57] Weed D.L. (1991). The merger of bioethics and epidemiology. J Clin Epidemiol.

[58] Virtanen A., Spinelli F.R., Telliez J.B., O’Shea J.J., Silvennoinen O., Gadina M. (2024). JAK inhibitor selectivity: new opportunities, better drugs?. Nat Rev Rheumatol.

[59] de Coupade C., Ajuebor M.N., Russo-Marie F., Perretti M., Solito E. (2001). Cytokine modulation of liver annexin 1 expression during experimental endotoxemia. Am J Pathol.

[60] Perretti M., Chiang N., La M. (2002). Endogenous lipid- and peptide-derived anti-inflammatory pathways generated with glucocorticoid and aspirin treatment activate the lipoxin A4 receptor. Nat Med.

[61] Serhan C.N., Yang R., Martinod K. (2009). Maresins: novel macrophage mediators with potent antiinflammatory and proresolving actions. J Exp Med.

[62] Cash J.L., Christian A.R., Greaves D.R. (2010). Chemerin peptides promote phagocytosis in a ChemR23- and Syk-dependent manner. J Immunol.

[63] Getting S.JK., Flower R.J., Perretti M. (1997). Inhibition of neutrophil and monocyte recruitment by endogenous and exogenous lipocortin 1. Br J Pharmacol.

[64] Schwab J.M., Chiang N., Arita M., Serhan C.N. (2007). Resolvin E1 and protectin D1 activate inflammation-resolution programmes. Nature.

[65] Kolaczkowska E., Koziol A., Plytycz B., Arnold B. (2010). Inflammatory macrophages, and not only neutrophils, die by apoptosis during acute peritonitis. Immunobiology.

[66] Yamada T., Tani Y., Nakanishi H., Taguchi R., Arita M., Arai H. (2011). Eosinophils promote resolution of acute peritonitis by producing proresolving mediators in mice. FASEB J.

[67] Lopez-Castejón G., Baroja-Mazo A., Pelegrín P. (2011). Novel macrophage polarization model: from gene expression to identification of new anti-inflammatory molecules. Cell Mol Life Sci.

[68] Devchand P.R., Arita M., Hong S. (2003). Human ALX receptor regulates neutrophil recruitment in transgenic mice: roles in inflammation and host defense. FASEB J.

[69] Fredman G., Li Y., Dalli J., Chiang N., Serhan C.N. (2012). Self-limited versus delayed resolution of acute inflammation: temporal regulation of pro-resolving mediators and microRNA. Sci Rep.

[70] Bannenberg G., Moussignac R.L., Gronert K. (2004). Lipoxins and novel 15-epi-lipoxin analogs display potent anti-inflammatory actions after oral administration. Br J Pharmacol.

[71] Montero-Melendez T., Patel H.B., Seed M., Nielsen S., Jonassen T.E.N., Perretti M. (2011). The melanocortin agonist AP214 exerts anti-inflammatory and proresolving properties. Am J Pathol.

[72] Montero-Melendez T., Gobbetti T., Cooray S.N., Jonassen T.E.N., Perretti M. (2015). Biased agonism as a novel strategy to harness the proresolving properties of melanocortin receptors without eliciting melanogenic effects. J Immunol.

[73] de Gaetano M., Tighe C., Gahan K. (2021). Asymmetric synthesis and biological screening of quinoxaline-containing synthetic lipoxin A_4_ mimetics (QNX-sLXms). J Med Chem.

[74] Bannenberg G.L., Chiang N., Ariel A. (2005). Molecular circuits of resolution: formation and actions of resolvins and protectins. J Immunol.

[75] Galvão I., Melo E.M., de Oliveira V.L.S. (2021). Therapeutic potential of the FPR2/ALX agonist AT-01-KG in the resolution of articular inflammation. Pharmacol Res.

[76] Garrido-Mesa J., Thomas B.L., Dodd J., Spana C., Perretti M., Montero-Melendez T. (2022). Pro-resolving and anti-arthritic properties of the MC_1_ selective agonist PL8177. Front Immunol.

[77] Choi M.Y., Cook N.R., Kotler G., Serhan C.N., Tatituri R., Costenbader K.H. (2022). Fish oil supplementation in patients with and without systemic lupus erythematosus: targeting pro-inflammatory and pro-resolving lipid mediators. Clin Exp Rheumatol.

[78] Bento A.F., Claudino R.F., Dutra R.C., Marcon R., Calixto J.B. (2011). Omega-3 fatty acid-derived mediators 17(R)-hydroxy docosahexaenoic acid, aspirin-triggered resolvin D1 and resolvin D2 prevent experimental colitis in mice. J Immunol.

[79] Brennan E.P., Mohan M., McClelland A. (2018). Lipoxins regulate the early growth Response-1 network and reverse diabetic kidney disease. J Am Soc Nephrol.

[80] Yin Y., Chen F., Wang W., Wang H., Zhang X. (2017). Resolvin D1 inhibits inflammatory response in STZ-induced diabetic retinopathy rats: possible involvement of NLRP3 inflammasome and NF-κB signaling pathway. Mol Vis.

[81] Tang Y., Zhang M.J., Hellmann J., Kosuri M., Bhatnagar A., Spite M. (2013). Proresolution therapy for the treatment of delayed healing of diabetic wounds. Diabetes.

[82] Xu J., Li H.B., Chen L. (2019). BML-111 accelerates the resolution of inflammation by modulating the Nrf2/HO-1 and NF-κB pathways in rats with ventilator-induced lung injury. Int Immunopharmacol.

[83] He M., Cheng N., Gao W.W. (2011). Characterization of Quin-C1 for its anti-inflammatory property in a mouse model of bleomycin-induced lung injury. Acta Pharmacol Sin.

[84] El Kebir D., Gjorstrup P., Filep J.G. (2012). Resolvin E1 promotes phagocytosis-induced neutrophil apoptosis and accelerates resolution of pulmonary inflammation. Proc Natl Acad Sci U S A.

[85] Karra L., Haworth O., Priluck R., Levy B.D., Levi-Schaffer F. (2015). Lipoxin B_4_ promotes the resolution of allergic inflammation in the upper and lower airways of mice. Mucosal Immunol.

[86] Haworth O., Cernadas M., Yang R., Serhan C.N., Levy B.D. (2008). Resolvin E1 regulates interleukin 23, interferon-gamma and lipoxin A4 to promote the resolution of allergic airway inflammation. Nat Immunol.

[87] Gerlach B.D., Marinello M., Heinz J. (2020). Resolvin D1 promotes the targeting and clearance of necroptotic cells. Cell Death Differ.

[88] Bardin M., Pawelzik S.C., Lagrange J. (2022). The resolvin D2—GPR18 axis is expressed in human coronary atherosclerosis and transduces atheroprotection in apolipoprotein E deficient mice. Biochem Pharmacol.

[89] Petri M.H., Laguna-Fernandez A., Arnardottir H. (2017). Aspirin-triggered lipoxin A4 inhibits atherosclerosis progression in apolipoprotein E^-/-^ mice. Br J Pharmacol.

[90] Chen J., Oggero S., Cecconello C. (2023). The annexin-A1 mimetic RTP-026 promotes acute cardioprotection through modulation of immune cell activation. Pharmacol Res.

[91] Chen J., Purvis G.S.D., Collotta D. (2020). RvE1 attenuates polymicrobial sepsis-induced cardiac dysfunction and enhances bacterial clearance. Front Immunol.

[92] Al-Shaer A.E., Pal A., Shi Q. (2022). Modeling human heterogeneity of obesity with diversity outbred mice reveals a fat mass-dependent therapeutic window for resolvin E1. FASEB J.

[93] Fredman G., Serhan C.N. (2024). Specialized pro-resolving mediators in vascular inflammation and atherosclerotic cardiovascular disease. Nat Rev Cardiol.

[94] Krashia P., Cordella A., Nobili A. (2019). Blunting neuroinflammation with resolvin D1 prevents early pathology in a rat model of Parkinson’s disease. Nat Commun.

[95] Kantarci A., Aytan N., Palaska I. (2018). Combined administration of resolvin E1 and lipoxin A4 resolves inflammation in a murine model of Alzheimer’s disease. Exp Neurol.

[96] Yildiz O., Hunt G.P., Schroth J. (2025). Lipid-mediated resolution of inflammation and survival in amyotrophic lateral sclerosis. Brain Commun.

[97] Kim J., Joshi H.P., Sheen S.H. (2021). Resolvin D3 promotes inflammatory resolution, neuroprotection, and functional recovery after spinal cord injury. Mol Neurobiol.

[98] Yang T., Xu G., Newton P.T. (2019). Maresin 1 attenuates neuroinflammation in a mouse model of perioperative neurocognitive disorders. Br J Anaesth.

[99] Livshits G., Kalinkovich A. (2022). Specialized, pro-resolving mediators as potential therapeutic agents for alleviating fibromyalgia symptomatology. Pain Med.

[100] Nesman J.I., Chen O., Luo X., Ji R.R., Serhan C.N., Hansen T.V. (2021). A new synthetic protectin D1 analog 3-oxa-PD1_n-3 DPA_ reduces neuropathic pain and chronic itch in mice. Org Biomol Chem.

[101] Hamlett E.D., Hjorth E., Ledreux A., Gilmore A., Schultzberg M., Granholm A.C. (2020). RvE1 treatment prevents memory loss and neuroinflammation in the Ts65Dn mouse model of Down syndrome. Glia.

[102] Frigerio F., Pasqualini G., Craparotta I. (2018). n-3 docosapentaenoic acid-derived protectin D1 promotes resolution of neuroinflammation and arrests epileptogenesis. Brain.

[103] Sánchez-Fernández A., Zandee S., Mastrogiovanni M. (2022). Administration of Maresin-1 ameliorates the physiopathology of experimental autoimmune encephalomyelitis. J Neuroinflammation.

[104] Madeira M.F.M., Queiroz-Junior C.M., Montero-Melendez T. (2016). Melanocortin agonism as a viable strategy to control alveolar bone loss induced by oral infection. FASEB J.

[105] Wu Y.C., Yu N., Rivas C.A., Mehrnia N., Kantarci A., Van Dyke T.E. (2023). RvE1 promotes Axin2+ cell regeneration and reduces bacterial invasion. J Dent Res.

[106] Hasturk H., Schulte F., Martins M. (2021). Safety and preliminary efficacy of a novel host-modulatory therapy for reducing gingival inflammation. Front Immunol.

[107] Sansbury B.E., Li X., Wong B. (2021). PCTR1 enhances repair and bacterial clearance in skin wounds. Am J Pathol.

[108] Carion T.W., Kracht D., Strand E. (2019). VIP modulates the ALX/FPR2 receptor axis toward inflammation resolution in a mouse model of bacterial keratitis. Prostaglandins Other Lipid Mediat.

[109] Gracia Aznar A., Moreno Egea F., Gracia Banzo R. (2024). Pro-resolving inflammatory effects of a marine oil enriched in specialized pro-resolving mediators (SPMs) supplement and its implication in patients with post-COVID syndrome (PCS). Biomedicines.

[110] Almeida P.R.J., Periard A.M., Tana F.L. (2024). Effects of a pro-resolving drug in COVID-19: preclinical studies to a randomized, placebo-controlled, phase Ib/IIa trial in hospitalized patients. Br J Pharmacol.

[111] Isopi E., Mattoscio D., Codagnone M. (2020). Resolvin D1 reduces lung infection and inflammation activating resolution in cystic fibrosis. Front Immunol.

[112] Serhan C.N., Gupta S.K., Perretti M. (2020). The atlas of inflammation resolution (AIR). Mol Aspects Med.

[113] Munck A., Guyre P.M., Holbrook N.J. (1984). Physiological functions of glucocorticoids in stress and their relation to pharmacological actions. Endocr Rev.

[114] Morand E.F., Hall P., Hutchinson P., Yang Y.H. (2006). Regulation of annexin I in rheumatoid synovial cells by glucocorticoids and interleukin-1. Mediators Inflamm.

[115] Smith S.F., Tetley T.D., Guz A., Flower R.J. (1990). Detection of lipocortin 1 in human lung lavage fluid: lipocortin degradation as a possible proteolytic mechanism in the control of inflammatory mediators and inflammation. Environ Health Perspect.

[116] Bensalem N., Ventura A.P., Vallée B. (2005). Down-regulation of the anti-inflammatory protein annexin A1 in cystic fibrosis knock-out mice and patients. Mol Cell Proteomics.

[117] Granell R., Curtin J.A., Haider S. (2023). A meta-analysis of genome-wide association studies of childhood wheezing phenotypes identifies ANXA1 as a susceptibility locus for persistent wheezing. eLife.

[118] Costa V.V., Sugimoto M.A., Hubner J. (2022). Targeting the annexin A1-FPR2/ALX pathway for host-directed therapy in dengue disease. eLife.

[119] Weng H., Peng Y., Pei Q., Jing F., Yang M., Yi Q. (2021). Decreased serum annexin A1 levels in Kawasaki disease with coronary artery aneurysm. Pediatr Res.

[120] Särndahl E., Bergström I., Nijm J., Forslund T., Perretti M., Jonasson L. (2010). Enhanced neutrophil expression of annexin-1 in coronary artery disease. Metabolism.

[121] Bergström I., Lundberg A.K., Jönsson S., Särndahl E., Ernerudh J., Jonasson L. (2017). Annexin A1 in blood mononuclear cells from patients with coronary artery disease: its association with inflammatory status and glucocorticoid sensitivity. PLoS One.

[122] Viiri L.E., Full L.E., Navin T.J. (2013). Smooth muscle cells in human atherosclerosis: proteomic profiling reveals differences in expression of annexin A1 and mitochondrial proteins in carotid disease. J Mol Cell Cardiol.

[123] Zhou C., Lin Z., Cao H. (2022). Anxa1 in smooth muscle cells protects against acute aortic dissection. Cardiovasc Res.

[124] Ramiro L., García-Berrocoso T., Briansó F. (2021). Integrative multi-omics analysis to characterize human brain ischemia. Mol Neurobiol.

[125] Adel F.W., Rikhi A., Wan S.H. (2020). Annexin A1 is a potential novel biomarker of congestion in acute heart failure. J Card Fail.

[126] Locatelli I., Sutti S., Jindal A. (2014). Endogenous annexin A1 is a novel protective determinant in nonalcoholic steatohepatitis in mice. Hepatology.

[127] Gadipudi L.L., Ramavath N.N., Provera A. (2022). Annexin A1 treatment prevents the evolution to fibrosis of experimental nonalcoholic steatohepatitis. Clin Sci.

[128] Lin S., Liang F., Chen C. (2025). Annexin A1 regulates inflammatory-immune response and reduces pancreatic and extra- pancreatic injury during severe acute pancreatitis. Genes Immun.

[129] Cristante E., McArthur S., Mauro C. (2013). Identification of an essential endogenous regulator of blood-brain barrier integrity, and its pathological and therapeutic implications. Proc Natl Acad Sci U S A.

[130] Qin C.X., Norling L.V., Vecchio E.A., Qin C.X., Norling L.V., Vecchio E.A. (2022). Formylpeptide receptor 2: nomenclature, structure, signalling and translational perspectives: IUPHAR review 35. Br J Pharmacol.

[131] Simiele F., Recchiuti A., Mattoscio D. (2012). Transcriptional regulation of the human FPR2/ALX gene: evidence of a heritable genetic variant that impairs promoter activity. FASEB J.

[132] Salemi M., Schillaci F.A., Lanza G. (2024). Transcriptome study in Sicilian patients with autism spectrum disorder. Biomedicines.

[133] Perretti M., Dalli J. (2023). Resolution pharmacology: focus on pro-resolving annexin A1 and lipid mediators for therapeutic innovation in inflammation. Annu Rev Pharmacol Toxicol.

[134] Dalli J., Consalvo A.P., Ray V. (2013). Proresolving and tissue-protective actions of annexin A1-based cleavage-resistant peptides are mediated by formyl peptide receptor 2/lipoxin A4 receptor. J Immunol.

[135] Zhang S., Gong H., Ge Y., Ye R.D. (2020). Biased allosteric modulation of formyl peptide receptor 2 leads to distinct receptor conformational states for pro- and anti-inflammatory signaling. Pharmacol Res.

[136] Ge Y., Zhang S., Wang J. (2020). Dual modulation of formyl peptide receptor 2 by aspirin-triggered lipoxin contributes to its anti-inflammatory activity. FASEB J.

[137] Maciuszek M., Cacace A., Brennan E., Godson C., Chapman T.M. (2021). Recent advances in the design and development of formyl peptide receptor 2 (FPR2/ALX) agonists as pro-resolving agents with diverse therapeutic potential. Eur J Med Chem.

[138] Stalder A.K., Lott D., Strasser D.S. (2017). Biomarker-guided clinical development of the first-in-class anti-inflammatory FPR2/ALX agonist ACT-389949. Br J Clin Pharmacol.

[139] García R.A., Lupisella J.A., Ito B.R. (2021). Selective FPR2 agonism promotes a proresolution macrophage phenotype and improves cardiac structure-function post myocardial infarction. JACC Basic Transl Sci.

[140] Asahina Y., Wurtz N.R., Arakawa K. (2020). Discovery of BMS-986235/LAR-1219: A potent formyl peptide receptor 2 (FPR2) selective agonist for the prevention of heart failure. J Med Chem.

[141] Lupisella J., St-Onge S., Carrier M. (2022). Molecular mechanisms of desensitization underlying the differential effects of formyl peptide receptor 2 agonists on cardiac structure-function post myocardial infarction. ACS Pharmacol Transl Sci.

[142] Godson C., Guiry P., Brennan E. (2023). Lipoxin mimetics and the resolution of inflammation. Annu Rev Pharmacol Toxicol.

[143] Singh J., Jackson K.L., Fang H. (2024). Novel formylpeptide receptor 1/2 agonist limits hypertension-induced cardiovascular damage. Cardiovasc Res.

[144] Margraf A., Chen J., Christoforou M. (2025). Formyl-peptide receptor type 2 activation mitigates heart and lung damage in inflammatory arthritis. EMBO Mol Med.

[145] Maurer J., de Groot A., Martin L., Grouzmann E., Wuerzner G., Eugster P.J. (2024). Quantification of endogenous angiotensin 1–10, 1–9, 1–8, 1–7, and 1–5 in human plasma using micro-UHPLC-MS/MS: outlining the importance of the pre-analytics for reliable results. J Pharm Biomed Anal.

[146] Haschke M., Schuster M., Poglitsch M. (2013). Pharmacokinetics and pharmacodynamics of recombinant human angiotensin-converting enzyme 2 in healthy human subjects. Clin Pharmacokinet.

[147] da Silveira K.D., Coelho F.M., Vieira A.T. (2010). Anti-inflammatory effects of the activation of the angiotensin-(1-7) receptor, MAS, in experimental models of arthritis. J Immunol.

[148] da Silveira K.D., Pompermayer Bosco K.S., Diniz L.R.L. (2010). ACE2-angiotensin-(1–7)-Mas axis in renal ischaemia/reperfusion injury in rats. Clin Sci.

[149] Barroso L.C., Magalhaes G.S., Galvão I. (2017). Angiotensin-(1-7) promotes resolution of neutrophilic inflammation in a model of antigen-induced arthritis in mice. Front Immunol.

[150] Magalhaes G.S., Barroso L.C., Reis A.C. (2018). Angiotensin-(1-7) promotes resolution of eosinophilic inflammation in an experimental model of asthma. Front Immunol.

[151] Saravi B., Li Z., Lang C.N. (2021). The tissue renin-angiotensin system and its role in the pathogenesis of major human diseases: quo vadis?. Cells.

[152] Queiroz-Junior C.M., Santos A.C.P.M., Galvão I. (2019). The angiotensin converting enzyme 2/angiotensin-(1-7)/Mas Receptor axis as a key player in alveolar bone remodeling. Bone.

[153] Saravi B., Lang G., Ülkümen S. (2020). The tissue renin-angiotensin system (tRAS) and the impact of its inhibition on inflammation and bone loss in the periodontal tissue. Eur Cell Mater.

[154] Becker L.K., Totou N., Moura S. (2018). Eccentric overload muscle damage is attenuated by a novel angiotensin- (1–7) treatment. Int J Sports Med.

[155] Rodgers K.E., Bolton L.L., Verco S., diZerega G.S. (2015). NorLeu^3^-angiotensin (1-7) [DSC127] as a therapy for the healing of diabetic foot ulcers. Adv Wound Care.

[156] Nguyen T.T., Ding D., Wolter W.R. (2018). Expression of active matrix metalloproteinase-9 as a likely contributor to the clinical failure of aclerastide in treatment of diabetic foot ulcers. Eur J Pharmacol.

[157] Nocchi E., Scalzo S., Rocha-Resende C. (2024). The Mas agonist CGEN-856S prevents Ang II induced cardiomyocyte hypertrophy via nitric oxide production. Peptides.

[158] Deng Y., Ding W., Peng Q., Wang W., Duan R., Zhang Y. (2024). Advancement in beneficial effects of AVE 0991: a brief review. Mini Rev Med Chem.

[159] Cone R.D., Mountjoy K.G., Robbins L.S. (1993). Cloning and functional characterization of a family of receptors for the melanotropic peptides. Ann N Y Acad Sci.

[160] Montero-Melendez T. (2015). ACTH: the forgotten therapy. Semin Immunol.

[161] Herpin T.F., Yu G., Carlson K.E. (2003). Discovery of tyrosine-based potent and selective melanocortin-1 receptor small-molecule agonists with anti-inflammatory properties. J Med Chem.

[162] Montero-Melendez T., Nagano A., Chelala C., Filer A., Buckley C.D., Perretti M. (2020). Therapeutic senescence via GPCR activation in synovial fibroblasts facilitates resolution of arthritis. Nat Commun.

[163] Yu S., Doycheva D.M., Gamdzyk M. (2021). Activation of MC1R with BMS-470539 attenuates neuroinflammation via cAMP/PKA/Nurr1 pathway after neonatal hypoxic-ischemic brain injury in rats. J Neuroinflammation.

[164] Gesualdo C., Balta C., Platania C.B.M. (2021). Fingolimod and diabetic retinopathy: a drug repurposing study. Front Pharmacol.

[165] Can V.C., Locke I.C., Kaneva M.K. (2020). Novel anti-inflammatory and chondroprotective effects of the human melanocortin MC1 receptor agonist BMS-470539 dihydrochloride and human melanocortin MC3 receptor agonist PG-990 on lipopolysaccharide activated chondrocytes. Eur J Pharmacol.

[166] Jang E.A., Kim J.Y., Tin T.D., Song J.A., Lee S.H., Kwak S.H. (2019). The effects of BMS-470539 on lipopolysaccharide-induced acute lung injury. Acute Crit Care.

[167] Fan K., Zeng L., Guo J. (2021). Visualized podocyte-targeting and focused ultrasound responsive glucocorticoid nano-delivery system against immune-associated nephropathy without glucocorticoid side effect. Theranostics.

[168] Catania A., Airaghi L., Garofalo L., Cutuli M., Lipton J.M. (1998). The neuropeptide α-MSH in HIV infection and other disorders in humans. Ann N Y Acad Sci.

[169] Roth C.L., Enriori P.J., Gebhardt U. (2010). Changes of peripheral α-melanocyte-stimulating hormone in childhood obesity. Metabolism.

[170] Mao Z., Liu G., Chen J.J. (2018). Serum *α*-melanocyte-stimulating hormone may act as a protective biomarker for non-traumatic osteonecrosis of the femoral head. Ann Clin Biochem.

[171] Candemir B., İleri İ., Yalçın M.M. (2023). Relationship between appetite-related peptides and frailty in older adults. Endocr Res.

[172] Zierath D., Tanzi P., Cain K., Shibata D., Becker K. (2011). Plasma α-melanocyte stimulating hormone predicts outcome in ischemic stroke. Stroke.

[173] Vidojevic D., Seman S., Lasica R. (2021). Alpha-melanocyte-stimulating hormone during exercise recovery has prognostic value for coronary artery disease. Hormones.

[174] Catania A., Gerloni V., Procaccia S. (1994). The anticytokine neuropeptide α-melanocyte-stimulating hormone in synovial fluid of patients with rheumatic diseases: comparisons with other anticytokine molecules. Neuroimmunomodulation.

[175] Fontaine T., Busch A., Laeremans T. (2024). Structure elucidation of a human melanocortin-4 receptor specific orthosteric nanobody agonist. Nat Commun.

[176] Tafreshi N.K., Tichacek C.J., Pandya D.N. (2019). Melanocortin 1 receptor-targeted *α*-particle therapy for metastatic uveal melanoma. J Nucl Med.

[177] Li M., Liu D., Lee D. (2021). Targeted alpha-particle radiotherapy and immune checkpoint inhibitors induces cooperative inhibition on tumor growth of malignant melanoma. Cancers.

[178] Langendonk J.G., Balwani M., Anderson K.E. (2015). Afamelanotide for erythropoietic protoporphyria. N Engl J Med.

[179] Evans D., Kenyon K., Ousler G. (2023). Efficacy and safety of the melanocortin pan-agonist PL9643 in a Phase 2 study of patients with dry eye disease. J Ocul Pharmacol Ther.

[180] Dodd J., Jordan R., Makhlina M. (2023). A novel oral formulation of the melanocortin-1 receptor agonist PL8177 resolves inflammation in preclinical studies of inflammatory bowel disease and is gut restricted in rats, dogs, and humans. Front Immunol.

[181] Lindskog A., Ebefors K., Johansson M.E. (2010). Melanocortin 1 receptor agonists reduce proteinuria. J Am Soc Nephrol.

[182] Montero-Melendez T., Boesen T., Jonassen T.E.N. (2022). Translational advances of melanocortin drugs: integrating biology, chemistry and genetics. Semin Immunol.

[183] Haskó G., Cronstein B.N. (2004). Adenosine: an endogenous regulator of innate immunity. Trends Immunol.

[184] Martin C., Leone M., Viviand X., Ayem M.L., Guieu R. (2000). High adenosine plasma concentration as a prognostic index for outcome in patients with septic shock. Crit Care Med.

[185] Sottofattori E., Anzaldi M., Ottonello L. (2001). HPLC determination of adenosine in human synovial fluid. J Pharm Biomed Anal.

[186] Montesinos M.C., Gadangi P., Longaker M. (1997). Wound healing is accelerated by agonists of adenosine A2 (G α s-linked) receptors. J Exp Med.

[187] Rubenich D.S., de Souza P.O., Omizzollo N., Lenz G.S., Sevigny J., Braganhol E. (2021). Neutrophils: fast and furious-the nucleotide pathway. Purinergic Signal.

[188] Xu K., Cooney K.A., Shin E.Y. (2019). Adenosine from a biologic source regulates neutrophil extracellular traps (NETs). J Leukoc Biol.

[189] Riff R., Naamani O., Mazar J., Haviv Y.S., Chaimovitz C., Douvdevani A. (2021). A_1_ and A_2A_ adenosine receptors play a protective role to reduce prevalence of autoimmunity following tissue damage. Clin Exp Immunol.

[190] Fige É., Szendrei J., Sós L. (2021). Heme oxygenase-1 contributes to both the engulfment and the anti-inflammatory program of macrophages during efferocytosis. Cells.

[191] Sciaraffia E., Riccomi A., Lindstedt R. (2014). Human monocytes respond to extracellular cAMP through A2A and A2B adenosine receptors. J Leukoc Biol.

[192] Zhai Y.J., Liu P., He H.R. (2015). The association of ADORA2A and ADORA2B polymorphisms with the risk and severity of chronic heart failure: a case-control study of a northern Chinese population. Int J Mol Sci.

[193] Sohn R., Junker M., Meurer A., Zaucke F., Straub R.H., Jenei-Lanzl Z. (2021). Anti-inflammatory effects of endogenously released adenosine in synovial cells of osteoarthritis and rheumatoid arthritis patients. Int J Mol Sci.

[194] Valls M.D., Soldado M., Arasa J. (2021). Annexin A2-mediated plasminogen activation in endothelial cells contributes to the proangiogenic effect of adenosine A_2A_ Receptors. Front Pharmacol.

[195] Murray J.J., Weiler J.M., Schwartz L.B. (2009). Safety of Binodenoson, a selective adenosine A2a receptor agonist vasodilator pharmacological stress agent, in healthy subjects with mild intermittent asthma. Circ Cardiovasc Imaging.

[196] Buhr C., Gössl M., Erbel R., Eggebrecht H. (2008). Regadenoson in the detection of coronary artery disease. Vasc Health Risk Manag.

[197] Barrett R.J., Lamson M.J., Johnson J., Smith W.B. (2005). Pharmacokinetics and safety of Binodenoson after intravenous dose escalation in healthy volunteers. J Nucl Cardiol.

[198] Perretti M. (1997). Endogenous mediators that inhibit the leukocyte-endothelium interaction. Trends Pharmacol Sci.

[199] Godson C., Mitchell S., Harvey K., Petasis N.A., Hogg N., Brady H.R. (2000). Cutting edge: lipoxins rapidly stimulate nonphlogistic phagocytosis of apoptotic neutrophils by monocyte-derived macrophages. J Immunol.

[200] Gomez E.A., Colas R.A., Souza P.R. (2020). Blood pro-resolving mediators are linked with synovial pathology and are predictive of DMARD responsiveness in rheumatoid arthritis. Nat Commun.

[201] Barden A.E., Moghaddami M., Mas E., Phillips M., Cleland L.G., Mori T.A. (2016). Specialised pro-resolving mediators of inflammation in inflammatory arthritis. Prostaglandins Leukot Essent Fatty Acids.

[202] Alivernini S., MacDonald L., Elmesmari A. (2020). Distinct synovial tissue macrophage subsets regulate inflammation and remission in rheumatoid arthritis. Nat Med.

[203] Sano Y., Toyoshima S., Miki Y. (2020). Activation of inflammation and resolution pathways of lipid mediators in synovial fluid from patients with severe rheumatoid arthritis compared with severe osteoarthritis. Asia Pac Allergy.

[204] Jónasdóttir H.S., Brouwers H., Kwekkeboom J.C. (2017). Targeted lipidomics reveals activation of resolution pathways in knee osteoarthritis in humans. Osteoarthr Cartil.

[205] Kooij G., Troletti C.D., Leuti A. (2020). Specialized pro-resolving lipid mediators are differentially altered in peripheral blood of patients with multiple sclerosis and attenuate monocyte and blood-brain barrier dysfunction. Haematologica.

[206] Gündoğar H., Üstün K., Şenyurt S.Z., Özdemir E.Ç., Sezer U., Erciyas K. (2021). Gingival crevicular fluid levels of cytokine, chemokine, and growth factors in patients with periodontitis or gingivitis and periodontally healthy subjects: a cross-sectional multiplex study. Cent Eur J Immunol.

[207] Planagumà A., Kazani S., Marigowda G. (2008). Airway lipoxin A4 generation and lipoxin A4 receptor expression are decreased in severe asthma. Am J Respir Crit Care Med.

[208] Ringholz F.C., Buchanan P.J., Clarke D.T. (2014). Reduced 15-lipoxygenase 2 and lipoxin A4/leukotriene B4 ratio in children with cystic fibrosis. Eur Respir J.

[209] Soundararajan R., Maurin M.M., Rodriguez-Silva J. (2025). Integration of lipidomics with targeted, single cell, and spatial transcriptomics defines an unresolved pro-inflammatory state in colon cancer. Gut.

[210] Fosshaug L.E., Colas R.A., Anstensrud A.K. (2019). Early increase of specialized pro-resolving lipid mediators in patients with ST-elevation myocardial infarction. EBiomedicine.

[211] Schaller M.S., Chen M., Colas R.A. (2020). Treatment with a marine oil supplement alters lipid mediators and leukocyte phenotype in healthy patients and those with peripheral artery disease. J Am Heart Assoc.

[212] Mai N.T.H., Dobbs N., Phu N.H. (2018). A randomised double blind placebo controlled phase 2 trial of adjunctive aspirin for tuberculous meningitis in HIV-uninfected adults. eLife.

[213] Colas R.A., Nhat L.T.H., Thuong N.T.T. (2019). Proresolving mediator profiles in cerebrospinal fluid are linked with disease severity and outcome in adults with tuberculous meningitis. FASEB J.

[214] Serhan C.N., Levy B.D. (2018). Resolvins in inflammation: emergence of the pro-resolving superfamily of mediators. J Clin Invest.

[215] Serhan C.N., Levy B.D. (2025). Proresolving lipid mediators in the respiratory system. Annu Rev Physiol.

[216] Gomez E.A., De Matteis R., Udomjarumanee P., Munroe P.B., Dalli J. (2024). An LGR6 frameshift variant abrogates receptor expression on select leukocyte subsets and is associated with viral infections. Blood.

[217] Schottelius A.J., Giesen C., Asadullah K. (2002). An aspirin-triggered lipoxin A4 stable analog displays a unique topical anti-inflammatory profile. J Immunol.

[218] Gewirtz A.T., Collier-Hyams L.S., Young A.N. (2002). Lipoxin A4 analogs attenuate induction of intestinal epithelial proinflammatory gene expression and reduce the severity of dextran sodium sulfate-induced colitis. J Immunol.

[219] Guilford W.J., Bauman J.G., Skuballa W. (2004). Novel 3-oxa lipoxin A4 analogues with enhanced chemical and metabolic stability have anti-inflammatory activity in vivo. J Med Chem.

[220] Wu S.H., Chen X.Q., Liu B., Wu H.J., Dong L. (2013). Efficacy and safety of 15(R/S)-methyl-lipoxin A(4) in topical treatment of infantile eczema. Br J Dermatol.

[221] Kong X., Wu S.H., Zhang L., Chen X.Q. (2017). Pilot application of lipoxin A_4_ analog and lipoxin A_4_ receptor agonist in asthmatic children with acute episodes. Exp Ther Med.

[222] Cholkar K., Trinh H.M., Vadlapudi A.D., Wang Z., Pal D., Mitra A.K. (2015). Interaction studies of resolvin E1 analog (RX-10045) with efflux transporters. J Ocul Pharmacol Ther.

[223] Torricelli A.A., Santhanam A., Agrawal V., Wilson S.E. (2014). Resolvin E1 analog RX-10045 0.1% reduces corneal stromal haze in rabbits when applied topically after PRK. Mol Vis.

[224] Resolvyx Pharmaceuticals, Inc Announces Positive Data from Phase 2 Clinical Trial of the Resolvin RX-10045 in Patients with Dry Eye Syndrome [press release]. 2009. https://www.flagshippioneering.com/news/press-release/resolvyx-announces-positive-data-phase-2-clinical-trial-resolvin-rx-10045-patients-dry-ey.

[225] Arita M., Bianchini F., Aliberti J. (2005). Stereochemical assignment, antiinflammatory properties, and receptor for the omega-3 lipid mediator resolvin E1. J Exp Med.

[226] Trilleaud C., Gauttier V., Biteau K. (2021). Agonist anti-ChemR23 mAb reduces tissue neutrophil accumulation and triggers chronic inflammation resolution. Sci Adv.

[227] Norling L.V., Ly L., Dalli J. (2017). Resolving inflammation by using nutrition therapy: roles for specialized proresolving mediators. Curr Opin Clin Nutr Metab Care.

[228] Hulander E., Bärebring L., Turesson Wadell A. (2021). Diet intervention improves cardiovascular profile in patients with rheumatoid arthritis: results from the randomized controlled cross-over trial ADIRA. Nutr J.

[229] Proudman S.M., James M.J., Spargo L.D. (2015). Fish oil in recent onset rheumatoid arthritis: a randomised, double-blind controlled trial within algorithm-based drug use. Ann Rheum Dis.

[230] Choi G.Y., Calder P.C. (2024). The differential effects of eicosapentaenoic acid and docosahexaenoic acid on cardiovascular risk factors: an updated systematic review of randomized controlled trials. Front Nutr.

[231] Serhan C.N., Bäck M., Chiurchiù V. (2024). Expert consensus report on lipid mediators: role in resolution of inflammation and muscle preservation. FASEB J.

[232] Wolyniak M., Wlodarczyk M., Piscitelli F. (2024). Modulation of CB1 and CB2 receptors and endocannabinoid activity in inflammatory bowel diseases. J Physiol Pharmacol.

[233] Bala K., Porel P., Aran K.R. (2024). Emerging roles of cannabinoid receptor CB2 receptor in the central nervous system: therapeutic target for CNS disorders. Psychopharmacol.

[234] Grabon W., Ruiz A., Gasmi N. (2024). CB2 expression in mouse brain: from mapping to regulation in microglia under inflammatory conditions. J Neuroinflammation.

[235] Rakotoarivelo V., Mayer T.Z., Simard M., Flamand N., Di Marzo V. (2024). The impact of the CB_2_ cannabinoid receptor in inflammatory diseases: an update. Molecules.

[236] Pamplona F.A., Ferreira J., Menezes de Lima O. (2012). Anti-inflammatory lipoxin A4 is an endogenous allosteric enhancer of CB1 cannabinoid receptor. Proc Natl Acad Sci U S A.

[237] Peltner L.K., Gluthmann L., Börner F. (2023). Cannabidiol acts as molecular switch in innate immune cells to promote the biosynthesis of inflammation-resolving lipid mediators. Cell Chem Biol.

[238] Piomelli D., Mabou Tagne A. (2022). Endocannabinoid-based therapies. Annu Rev Pharmacol Toxicol.

[239] Watson L., Tullus K., Marks S.D., Holt R.C., Pilkington C., Beresford M.W. (2012). Increased serum concentration of sphingosine-1-phosphate in juvenile-onset systemic lupus erythematosus. J Clin Immunol.

[240] Morita Y., Sakai E., Isago H., Ono Y., Yatomi Y., Kurano M. (2024). Alterations in urinary ceramides, sphingoid bases, and their phosphates among patients with kidney disease. Front Nephrol.

[241] Burg N., Salmon J.E., Hla T. (2022). Sphingosine 1-phosphate receptor-targeted therapeutics in rheumatic diseases. Nat Rev Rheumatol.

[242] Tanaka Y., Kondo K., Ichibori A. (2020). Amiselimod, a sphingosine 1-phosphate receptor-1 modulator, for systemic lupus erythematosus: a multicenter, open-label exploratory study. Lupus.

[243] Niaudet C., Jung B., Kuo A. (2023). Therapeutic activation of endothelial sphingosine-1-phosphate receptor 1 by chaperone-bound S1P suppresses proliferative retinal neovascularization. EMBO Mol Med.

[244] Mantovani A., Garlanda C. (2023). Humoral innate immunity and acute-phase proteins. N Engl J Med.

[245] Ron D., Brasier A.R., Wright K.A., Habener J.F. (1990). The permissive role of glucocorticoids on interleukin-1 stimulation of angiotensinogen gene transcription is mediated by an interaction between inducible enhancers. Mol Cell Biol.

[246] Xavier A.M., Anunciato A.K.O., Rosenstock T.R., Glezer I. (2016). Gene expression control by glucocorticoid receptors during innate immune responses. Front Endocrinol.

[247] Turner A.M., Ficker J.H., Vianello A. (2025). Advancing the understanding and treatment of lung pathologies associated with alpha 1 antitrypsin deficiency. Ther Adv Respir Dis.

[248] Chapman K.R., Burdon J.G.W., Piitulainen E. (2015). Intravenous augmentation treatment and lung density in severe *α* 1 antitrypsin deficiency (RAPID): a randomised, double-blind, placebo-controlled trial. Lancet.

[249] Kaneva M.K., Greco K.V., Headland S.E. (2017). Identification of novel chondroprotective mediators in resolving inflammatory exudates. J Immunol.

[250] Kaneva M.K., Muley M.M., Krustev E. (2021). Alpha-1-antitrypsin reduces inflammation and exerts chondroprotection in arthritis. FASEB J.

[251] Khoshdel A., Forootan M., Afsharinasab M., Rezaian M., Abbasifard M. (2023). Assessment of the circulatory concentrations of cathepsin D, cathepsin K, and alpha-1 antitrypsin in patients with knee osteoarthritis. Ir J Med Sci.

[252] Awbrey B.J., Kuong S.J., MacNeil K.L., Wright M. (1993). The role of alpha-1-protease inhibitor (A1PI) in the inhibition of protease activity in human knee osteoarthritis. Agents Actions Suppl.

[253] Boeuf S., Steck E., Pelttari K. (2008). Subtractive gene expression profiling of articular cartilage and mesenchymal stem cells: serpins as cartilage-relevant differentiation markers. Osteoarthr Cartil.

[254] Wanner J., Subbaiah R., Skomorovska-Prokvolit Y. (2013). Proteomic profiling and functional characterization of early and late shoulder osteoarthritis. Arthritis Res Ther.

[255] Bai X., Bai A., Tomasicchio M. (2022). *α*1-antitrypsin binds to the glucocorticoid receptor with anti-inflammatory and antimycobacterial significance in macrophages. J Immunol.

[256] Bai X., Gao J., Guan X. (2024). Analysis of alpha-1-antitrypsin (AAT)-regulated, glucocorticoid receptor-dependent genes in macrophages reveals a novel host defense function of AAT. Physiol Rep.

[257] Vong L., D’Acquisto F., Pederzoli-Ribeil M. (2007). Annexin 1 cleavage in activated neutrophils: a pivotal role for proteinase 3. J Biol Chem.

[258] Vago J.P., Tavares L.P., Sugimoto M.A. (2016). Proresolving actions of synthetic and natural protease inhibitors are mediated by annexin A1. J Immunol.

[259] Sugimoto M.A., Ribeiro A.L.C., Costa B.R.C. (2017). Plasmin and plasminogen induce macrophage reprogramming and regulate key steps of inflammation resolution via annexin A1. Blood.

[260] Kunder M., Lakshmaiah V., Moideen Kutty A.V. (2022). Selective decrease in alpha1-antitrypsin levels in diabetic retinopathy: could the levels of it be playing a role in the pathophysiology of diabetic retinopathy?. Indian J Med Res.

[261] Lebenthal Y., Brener A., Hershkovitz E. (2019). A phase II, double-blind, randomized, placebo-controlled, multicenter study evaluating the efficacy and safety of alpha-1 antitrypsin (AAT) (Glassia®) in the treatment of recent-onset type 1 diabetes. Int J Mol Sci.

[262] Mullard A. (2024). Sanofi buys Inhibrx and drug for alpha-1 antitrypsin deficiency for US$1.7 billion. Nat Rev Drug Discov.

[263] Feldt J., Schicht M., Garreis F., Welss J., Schneider U.W., Paulsen F. (2019). Structure, regulation and related diseases of the actin-binding protein gelsolin. Expert Rev Mol Med.

[264] Osborn T.M., Verdrengh M., Stossel T.P., Tarkowski A., Bokarewa M. (2008). Decreased levels of the gelsolin plasma isoform in patients with rheumatoid arthritis. Arthritis Res Ther.

[265] Lee J., Sasaki F., Koike E. (2024). Gelsolin alleviates rheumatoid arthritis by negatively regulating NLRP3 inflammasome activation. Cell Death Differ.

[266] Feldt J., Schicht M., Welss J. (2022). Production and secretion of gelsolin by both human macrophage- and fibroblast-like synoviocytes and GSN modulation in the synovial fluid of patients with various forms of arthritis. Biomedicines.

[267] Lutaty A., Soboh S., Schif-Zuck S., Ariel A. (2020). Resolution-associated lactoferrin peptides limit LPS signaling and cytokine secretion from human macrophages. Int J Mol Sci.

[268] O’Neill L.A.J., Kishton R.J., Rathmell J. (2016). A guide to immunometabolism for immunologists. Nat Rev Immunol.

[269] Evans V.A., O’Neill L.A.J. (Published online July 29, 2025). Lessons from glucocorticoids, metformin, and dimethyl fumarate: could targeting immunometabolism lead to better anti-inflammatory therapies?. Annu Rev Pharmacol Toxicol.

[270] Auger J.P., Zimmermann M., Faas M. (2024). Metabolic rewiring promotes anti-inflammatory effects of glucocorticoids. Nature.

[271] Diskin C., Day E.A., Henry Ó.C., Toller-Kawahisa J.E., O’Neill L.A.J. (2023). 4-octyl itaconate and dimethyl fumarate induce secretion of the anti-inflammatory protein annexin A1 via NRF2. J Immunol.

[272] Fu H., Vuononvirta J., Fanti S. (2023). The glucose transporter 2 regulates CD8^+^ T cell function via environment sensing. Nat Metab.

[273] Certo M., Pontarini E., Gilbert S.G. (2025). Lactate signalling leads to aggregation of immune-inflammatory hotspots and SLC5A12 blockade promotes their resolution. Nat Metab.

[274] Rossi A.G., Sawatzky D.A., Walker A. (2006). Cyclin-dependent kinase inhibitors enhance the resolution of inflammation by promoting inflammatory cell apoptosis. Nat Med.

[275] Leitch A.E., Lucas C.D., Marwick J.A., Duffin R., Haslett C., Rossi A.G. (2012). Cyclin-dependent kinases 7 and 9 specifically regulate neutrophil transcription and their inhibition drives apoptosis to promote resolution of inflammation. Cell Death Differ.

[276] Meijer L., Hery-Arnaud G., Leven C. (2022). Safety and pharmacokinetics of Roscovitine (seliciclib) in cystic fibrosis patients chronically infected with Pseudomonas aeruginosa, a randomized, placebo-controlled study. J Cyst Fibros.

[277] Toussirot E., Bonnefoy F., Vauchy C., Perruche S., Saas P. (2021). Mini-review: the administration of apoptotic cells for treating rheumatoid arthritis: current knowledge and clinical perspectives. Front Immunol.

[278] Mevorach D., Zuckerman T., Reiner I. (2014). Single infusion of donor mononuclear early apoptotic cells as prophylaxis for graft-versus-host disease in myeloablative HLA-matched allogeneic bone marrow transplantation: a phase I/IIa clinical trial. Biol Blood Marrow Transplant.

[279] Mehrotra P., Ravichandran K.S. (2022). Drugging the efferocytosis process: concepts and opportunities. Nat Rev Drug Discov.

[280] Doran A.C. (2022). Inflammation resolution: implications for atherosclerosis. Circ Res.

[281] Jarr K.U., Nakamoto R., Doan B.H. (2021). Effect of CD47 blockade on vascular inflammation. N Engl J Med.

[282] Fredman G. (2019). DELineating resolution of inflammation. Nat Immunol.

[283] Kourtzelis I., Li X., Mitroulis I. (2019). DEL-1 promotes macrophage efferocytosis and clearance of inflammation. Nat Immunol.

[284] Dhawan U.K., Singhal A., Subramanian M. (2021). Dead cell and debris clearance in the atherosclerotic plaque: mechanisms and therapeutic opportunities to promote inflammation resolution. Pharmacol Res.

[285] Schett G., Neurath M.F. (2018). Resolution of chronic inflammatory disease: universal and tissue-specific concepts. Nat Commun.

[286] Rauber S., Luber M., Weber S. (2017). Resolution of inflammation by interleukin-9-producing type 2 innate lymphoid cells. Nat Med.

[287] da Costa J.C.S., Olsen P.C., de Azeredo Siqueira R. (2007). JMF2-1, a lidocaine derivative acting on airways spasm and lung allergic inflammation in rats. J Allergy Clin Immunol.

[288] Carvalho K.I.M., Coutinho D.S., Joca H.C. (2020). Anti-Bronchospasmodic effect of JME-173, a novel mexiletine analog endowed with highly attenuated anesthetic activity. Front Pharmacol.

[289] Norling L.V., Spite M., Yang R., Flower R.J., Perretti M., Serhan C.N. (2011). Cutting edge: humanized nano-proresolving medicines mimic inflammation-resolution and enhance wound healing. J Immunol.

[290] Thomas B.L., Montero-Melendez T., Oggero S. (2024). Molecular determinants of neutrophil extracellular vesicles that drive cartilage regeneration in inflammatory arthritis. Arthritis Rheumatol.

[291] Gauthier T., Martin-Rodriguez O., Chagué C. (2023). Amelioration of experimental autoimmune encephalomyelitis by in vivo reprogramming of macrophages using pro-resolving factors. J Neuroinflammation.

[292] Saas P., Vetter M., Maraux M., Bonnefoy F., Perruche S. (2022). Resolution therapy: harnessing efferocytic macrophages to trigger the resolution of inflammation. Front Immunol.

[293] Bhattacharya P., Dhawan U.K., Hussain M.T. (2023). Efferocytes release extracellular vesicles to resolve inflammation and tissue injury via prosaposin-GPR37 signaling. Cell Rep.

